# Chlorophyll Catabolites in Fall Leaves of the Wych Elm Tree Present a Novel Glycosylation Motif

**DOI:** 10.1002/chem.201601739

**Published:** 2016-06-06

**Authors:** Mathias Scherl, Thomas Müller, Christoph R. Kreutz, Roland G. Huber, Engelbert Zass, Klaus R. Liedl, Bernhard Kräutler

**Affiliations:** ^1^ Institute of Organic Chemistry and Center of Molecular BiosciencesUniversity of InnsbruckInnrain 80/826020InnsbruckAustria; ^2^ Laboratory of Organic ChemistryETH ZürichVladimir-Prelog-Weg 38093ZürichSwitzerland; ^3^ Institute of General, Inorganic & Theoretical Chemistry and Center of Molecular BiosciencesUniversity of InnsbruckInnrain 80/826020InnsbruckAustria; ^4^ Bioinformatics InstituteAgency for Science, Technology & Research30 Biopolis Street138671SingaporeSingapore

**Keywords:** chlorophyll, glycoside, natural products, plant senescence, tetrapyrrole

## Abstract

Fall leaves of the common wych elm tree (*Ulmus glabra*) were studied with respect to chlorophyll catabolites. Over a dozen colorless, non‐fluorescent chlorophyll catabolites (NCCs) and several yellow chlorophyll catabolites (YCCs) were identified tentatively. Three NCC fractions were isolated and their structures were characterized by spectroscopic means. Two of these, *Ug*‐NCC‐27 and *Ug*‐NCC‐43, carried a glucopyranosyl appendage. *Ug*‐NCC‐53, the least polar of these NCCs, was identified as the formal product of an intramolecular esterification of the propionate and primary glucopyranosyl hydroxyl groups of *Ug*‐NCC‐43. Thus, the glucopyranose moiety and three of the pyrrole units of *Ug*‐NCC‐53 span a 20‐membered ring, installing a bicyclo[17.3.1]glycoside moiety. This structural motif is unprecedented in heterocyclic natural products, according to a thorough literature search. The remarkable, three‐dimensional bicyclo[17.3.1]glycoside architecture reduces the flexibility of the linear tetrapyrrole. This feature of *Ug*‐NCC‐53 is intriguing, considering the diverse biological effects of known bicyclo[*n*.3.1]glycosidic natural products.

The annual disappearance of chlorophyll (Chl) has been a remarkable biological enigma until recently.^1^ In 1991, *Hv*‐NCC‐1 (**1 a**) was identified in senescent leaves of barley (*Hordeum vulgare*) as the first colorless linear tetrapyrrole derived from Chl.^2^ 1‐Formyl‐19‐oxobilane‐type tetrapyrroles, such as **1**, are now also classified as non‐fluorescent Chl catabolites (NCCs).^3^ Indeed, NCCs were found to accumulate in a variety of senescent leaves, apparently as “final” tetrapyrrolic products of a common pathway of the Chl‐breakdown (see Figure 1).^3^, ^4^, ^5^ However, in recent studies, the degradation of Chl was recognized to branch out, for example, to give persistent “hypermodified” fluorescent Chl‐catabolites (*hm*FCCs),^6^ as well as dioxobilin‐type NCCs (DNCCs).4b, 7 Dioxobilin‐type catabolites (such as DNCCs),7b–7d show a striking structural similarity to bilins, the abundant products of heme breakdown.^8^ Furthermore, yellow and pink Chl catabolites (YCCs and PiCCs, respectively) were found as apparent oxidation products of NCCs.^9^ In higher plants, Chl‐breakdown is, hence, revealed as producing a range of characteristic bilin‐type catabolites, named phyllobilins (Figure S1 in the Supporting Information),4b,4c, 5 all arising from the largely common PaO/phyllobilin pathway.^3^, ^4^, ^5^

**Figure 1:**
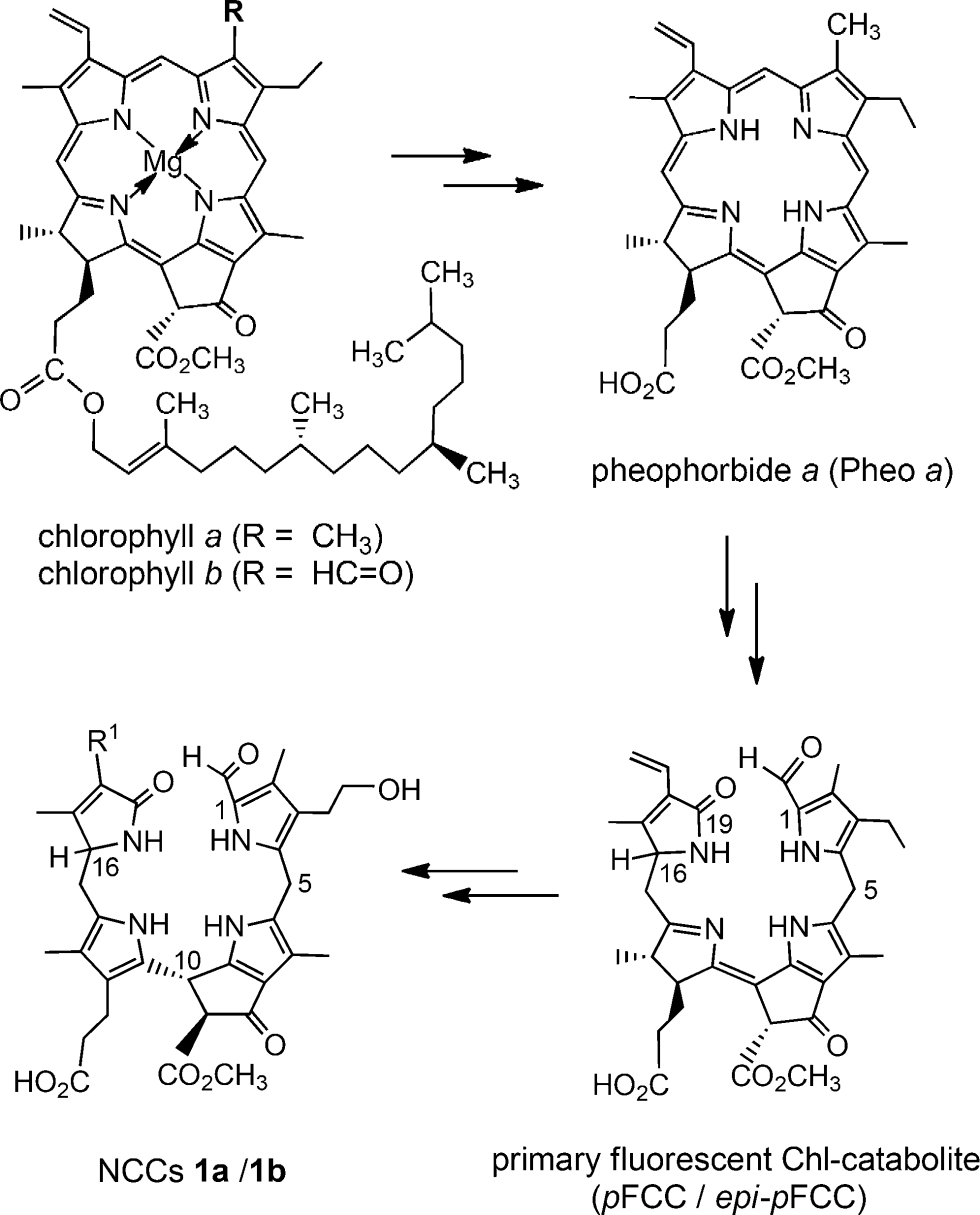
Structural outline of the chlorophyll breakdown to non‐fluorescing chlorophyll catabolites (NCCs) via the PaO/phyllobilin pathway.^5^ Pheophorbide *a* and primary fluorescing chlorophyll catabolites (*p*FCC/*epi*‐*p*FCC, two C 16‐epimers) are general intermediates. NCCs **1 a** (R^1^=CH(OH)CH_2_(OH)) and **1 b** (R^1^=CH=CH_2_) were first found in leaves of barley (*Hordeum vulgare*)^2^ or of the katsura tree (*Cercidipyhllum japonicum*),^11^ and were named *Hv*‐NCC‐1 and *Cj‐*NCC‐1, respectively.

Here, we describe an investigation of the Chl catabolites in senescent leaves of the wych elm tree (*Ulmus glabra*), a first representative of the genus *Ulmus* (*Ulmaceae*) to be studied in this respect. In this study, we discovered an NCC with a novel glycosylation topology. Wych elm is a widespread deciduous tree, occurring in mixed native forests in most parts of Europe. It grows as far north as Scotland and Finland, and as far to the south as Greece.^10^ The physiology of this Elm tree is of particular interest as it is threatened worldwide by the fungal dutch elm disease, transmitted by bark beetles (*Scolytus sp*.).^10^

Analysis of methanolic extracts of yellow (senescent) fall leaves of a wych elm tree by HPLC allowed the provisional identification of about a dozen colorless, non‐fluorescent Chl catabolites (NCCs), as well as several yellow Chl catabolites (YCCs), on the basis of their characteristic UV‐absorbance properties.^2^, ^12^ Three of the NCCs were isolated and further structurally characterized (Figure S2 A, B). From 115 g (wet weight) of senescent elm tree leaves, 7.1 mg (8.4 μmol) of *Ug*‐NCC‐27 (**2**), 12.2 mg (15.1 μmol) of *Ug*‐NCC‐43 (**3**), and 1.2 mg (1.5 μmol) of *Ug*‐NCC‐53 (**4**) were obtained, corresponding to about 63 % of the Chl in green leaves (for details see the Supporting Information).

The UV/Vis spectra of the *Ug*‐NCCs **2**–**4** displayed an absorbance near 312 nm, similar to those of **1 a** and **1 b** and other natural NCCs,^11^, ^13^ consistent with the presence of an α‐formylpyrrole moiety (Supporting Information and Figure S4, Figure S7 therein).^2^, 4a The CD spectra of the *Ug*‐NCCs **2**–**4** were also remarkably similar indicating a common configuration at C 10,4a as derived for **1 a**, **1 b** 11, 13 and other NCCs occurring in higher plants (Supporting Information and Figure S4).^14^ From mass spectra of *Ug*‐NCC‐27 (**2**) and *Ug*‐NCC‐43 (**3**), the molecular formulas were deduced as C_41_H_52_N_4_O_15_ and C_41_H_50_N_4_O_13_, respectively (Figure S6). Homo‐ and heteronuclear NMR spectra provided complete signal assignment for the two *Ug*‐NCCs, providing the basis for deducing their molecular constitutions (Supporting Information and Table S1). The ^1^H NMR spectra of *Ug*‐NCC‐27 (**2**) and of *Ug*‐NCC‐43 (**3**) showed signals typical of NCCs; in the spectrum of **3**, the characteristic signals for a vinyl group were present, but they were absent in the spectrum of **2**, in which a t‐like signal at 4.56 ppm was consistent with a dihydroxyethyl moiety. The constitutions of **2** and **3** turned out to be the same as those of some NCCs from other plants, for example, *Tc*‐NCC‐1 and *Tc*‐NCC‐2 from leaves of the lime tree (*Tilia cordata*).^15^ HPLC analysis (with co‐injection) of *Ug*‐NCC‐43 (**3**) and *Tc*‐NCC‐2 showed a common retention time (Supporting Information and Figure S9). This suggested that these two NCCs are identical, which also implies their common *epi*‐type configuration at C 16.^15^ In the course of the chlorophyll breakdown,5b the configuration of the NCCs at C 16 is installed at the stage of their FCC precursors by the enzyme red chlorophyll catabolite reductase (RCCR).5b, 16 RCCR of *Ulmus glabra* belongs to class‐2 RCCRs, which produce catabolites of the so‐called *epi*‐series.^17^

ESI‐MS analysis of *Ug*‐NCC‐53 (**4**) in the positiveion mode provided a [*M*+H]^+^‐ion signal at *m*/*z*=789.20 consistent with a *m*/*z*_calcd_ of [C_41_H_49_N_4_O_12_]^+^=789.33, furnishing the molecular formula of C_41_H_48_N_4_O_12_ (Figure S6). Fragment ions at *m*/*z*=757.20 and 666.27 indicated loss of MeOH (from the methyl ester functionality), as well as of C_7_H_9_NO (ring D), respectively. An ion was conspicuously absent that would correspond to the loss of C_6_H_10_O_5_ (the sugar moiety), as found in the spectra of **2** and **3** and typical of NCCs glucosylated at the 3^2^‐position. A 600 MHz ^1^H NMR spectrum of a solution of *Ug*‐NCC‐53 (**4**) in CD_3_OD (Figure 2) revealed signals of all 41 carbon‐bound protons, among them: a singlet (at low field) of the formyl proton, four singlets (at high field) of the four methyl groups attached at the β‐pyrrole positions, a singlet at 3.76 ppm (due to a methyl ester function), and the typical signal pattern for a vinyl group around 6 ppm.

**Figure 2:**
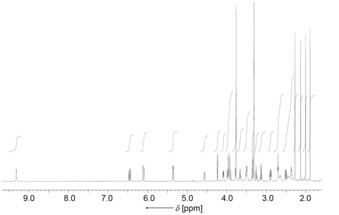
600 MHz ^1^H NMR spectrum of *Ug*‐NCC‐53 (**4**, CD_3_OD, 25 °C), see Table S1 in the Supporting Information for signal assignments.

^1^H,^1^H homonuclear correlations from ROESY spectra and ^1^H,^13^C‐heteronuclear correlations from HMBC spectra^18^ indicated a glucopyranosyl unit attached at C 3^2^ by an oxygen bridge to its anomeric carbon (Figure 3 and Figure S5 in the Supporting Information). The typical downfield shifts of the ^1^H and ^13^C signals for the methylene group H_2_C 3^2^ were also consistent with the peripheral sugar unit. Strikingly, ^1^H,^13^C‐heteronuclear correlations also showed a link of the primary oxygen at C 6′ of the glucopyranosyl moiety to the propionic side chain at ring C. Indeed, a significant shift to high field of the ^13^C signal of C 12^3^, compared to the position of the corresponding carboxyl carbon in NCCs with a free propionic side function (such as the *Ug*‐NCCs **2** and **3**), was consistent with the presence of an ester functionality (for details see the Supporting Information, Table S1). The signals of the ^1^H‐atoms at the C 5′‐ and C 6′‐positions of the sugar moiety of *Ug*‐NCC‐3 (**4**) were shifted to lower field, when compared to the spectrum of *Ug*‐NCC‐43 (**3**), also supporting the presence of an ester linkage at C 6′ of *Ug*‐NCC‐53 (Table S1). Conformational changes in the propionate chain are the likely cause for high‐field shifts of the ^13^C signals of C 12^1^ and C 12^2^. The bridging sugar moiety was identified as a β‐glucopyranosyl unit by comparing signal positions, ^1^H,^1^H‐coupling constants, and ^1^H,^1^H‐NOE correlations in the NMR spectra of **4** with the data of methyl‐β‐d‐glucopyranoside, β‐d‐glucose (Table S2),^19^ and with other NCCs with a peripheral β‐glucopyranosyl group at O 3^3^, for example, *Bn*‐NCC‐2^20^ or *Tc*‐NCC‐2.^15^ Remarkably, in the ^1^H NMR spectrum of *Ug*‐NCC‐53 (**4**) the signal of HC 8^2^ (at 3.78 ppm) disappeared only gradually during measurements in CD_3_OD, indicating slow H/D‐exchange at the α‐position of the β*‐*keto ester functionality. The proton at C 8^2^ coupled with HC 10 (d, *J*_H,H_=3.1 Hz), suggesting a relative *trans*‐configuration of HC 8^2^ and HC1 0, which is typical for stable C 8^2^ epimers of NCCs.14a An NOE correlation between the protons HC 10 and HC 5’ (of the NCC and glucose moieties, respectively) indicates that the bridging sugar is positioned close to the methine linker between rings B and C of the NCC **4**.

**Figure 3:**
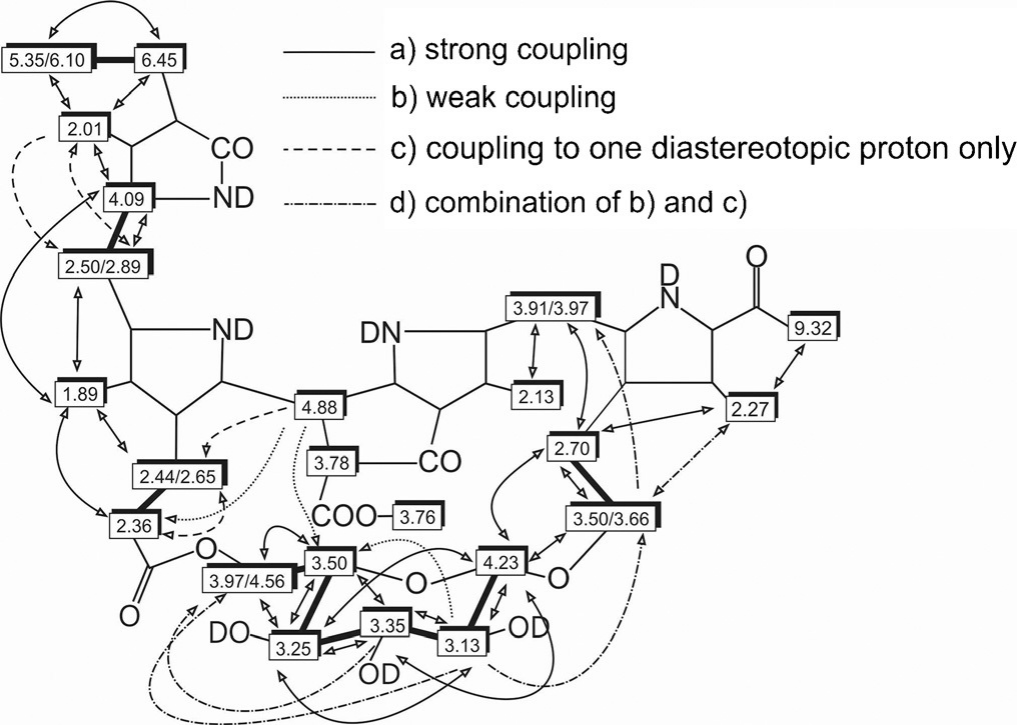
Graphical representations of ^1^H NMR chemical‐shift data of *Ug*‐NCC‐53 (**4**, in CD_3_OD, 25 °C) and NOE correlations (from ROESY spectra); bold lines indicate connectivities derived from the COSY spectra; arrows signify NOE correlations, classified as strong (full line) or medium (broken line).

The NCCs *Ug*‐NCC‐27 (**2**), *Ug*‐NCC‐43 (**3**), and *Ug*‐NCC‐53 (**4**) were all glycosylated at the C 3^2^‐position of ring A, as found in a variety of chlorophyll catabolites from higher plants.^4^, ^5^, ^15^ This suggests a broad metabolic significance to this type of functionalization with glucopyranosyl groups in Chl catabolites. Presumably the sugar units are attached to FCCs carrying an OH group at C 3^2^, and by enzymes in the cytosol that produce the corresponding glycosylated FCCs as direct NCC precursors (Figure 4).5b Indeed, the peripheral attachment of glucopyranosyl groups may be relevant for efficient trans‐membrane transport of FCCs and their deposition into the vacuoles, in which the highly stereoselective, non‐enzymatic FCC to NCC isomerisation14a is presumed to take place.5b, 21

**Figure 4:**
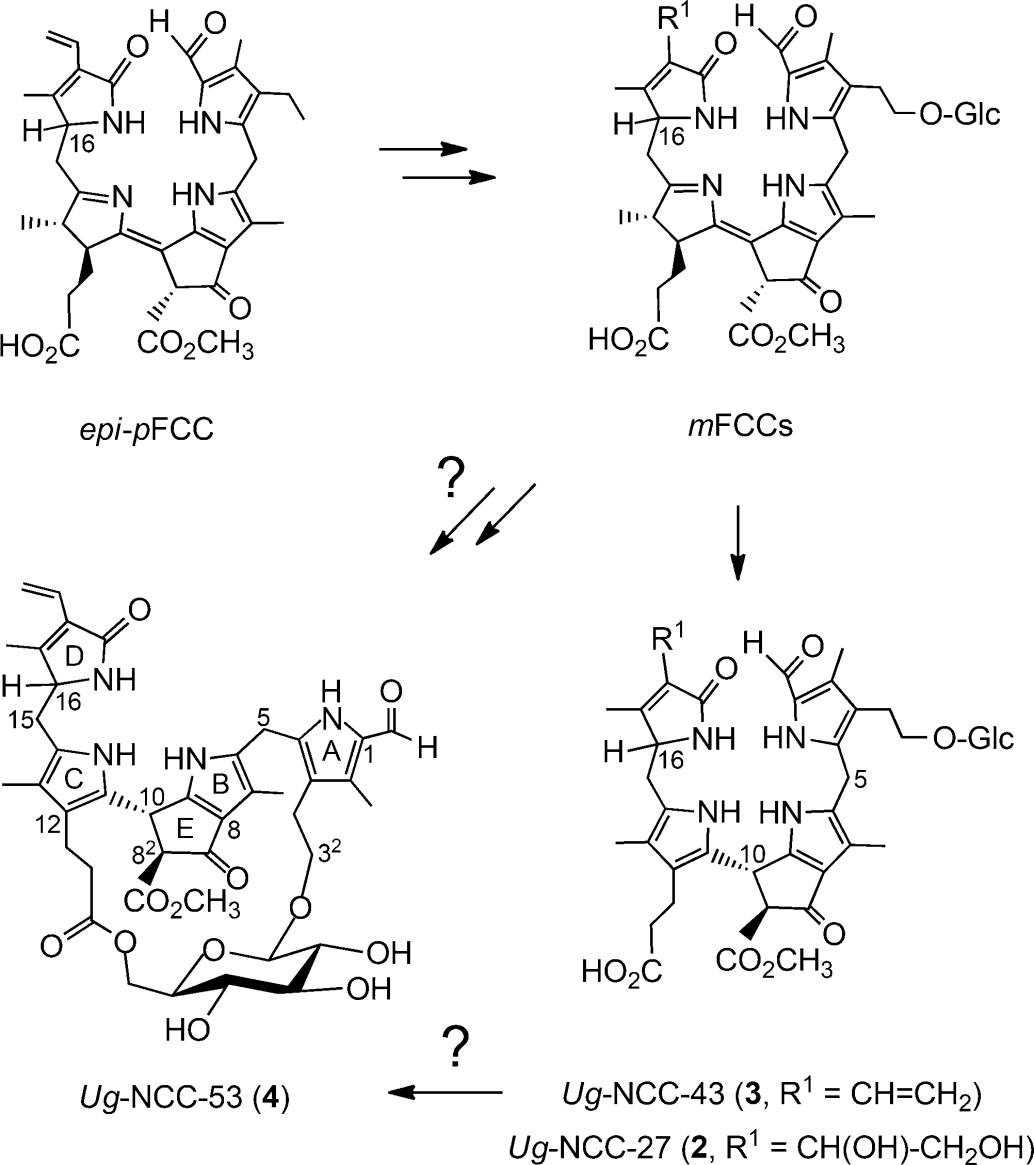
Possible late stages of the chlorophyll breakdown in senescent leaves of the wych elm tree. Hypothetical path from *epi*‐*p*FCC, via modified FCCs (*m*FCCs), to the three *Ug*‐NCCs **2**–**4** detected in the elm tree leaves (Glc=β‐d‐glucopyranose).

Terminal sugar units, as found in the NCCs **2** and **3**, as well as chain‐like or branched oligosaccharide moieties in glycosylated bio(macro)‐molecules decorate a multitude of natural products.^22^ However, in *Ug*‐NCC‐53 (**4**) the sugar moiety at ring A is also part of a striking bridging bicyclo[17.3.1]glycoside structure. The implied esterification of the sugar and the propionate group at ring C of this linear tetrapyrrole, clearly reduces the polarity of **4** compared to *Ug*‐NCC‐43 (**3**). Furthermore, by being part of a 20‐membered ring across the pyrrole rings A and C the bridging glycopyranosyl unit also imposes stringent structural restrictions onto the tetrapyrrole core of NCCs, which, otherwise, are conformationally flexible formyloxobilane‐type chlorophyll catabolites.

In view of the expected, relevant conformational effect of the unprecedented 1′‐6′‐glucopyranosyl bridge, the structure of *Ug*‐NCC‐53 (**4**) was analysed in molecular modelling studies (Figure 5). In these analyses a 1′‐6′‐β‐d‐glucopyranosyl bridge was used in the chair conformation, and both possible C 16‐epimers of **4** were taken into consideration, as well as the two (possible) epimers at C 10 (which were deduced indirectly to be *R* configurated14a). Thus, four stereoisomers were modelled with 10*R*/16*R*, 10*R*/16*S*, 10*R*/16*S* and 10*S*/16*S* configurations. Calculated distances were obtained using explicit solvent‐molecular‐dynamics simulations (see Experimental Section and Figure S11–14 in the Supporting Information). The calculated structure of the 10 R, 16 R‐isomer remained stable during the sampling time of 200 ns and displayed a short distance (3.0–3.5 Å) between HC 10 and HC 5′, consistent with the observation of an NOE correlation between these two H‐atoms in **4**. For the 10 R, 16 S‐isomer a short distance between HC 10 and HC 5’ was also calculated for the starting structure, which, however, underwent a reorganization after about 50 ns. In contrast, the calculated distance of HC 10 to HC 5’ for both 10 S‐isomers exceeded 7.5 Å. Hence, the modelling studies also supported the independently deduced *R*‐configuration at C 10 of the glycopyranosyl‐bridged NCC **4**.14a

**Figure 5:**
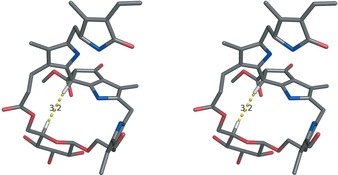
Stereoprojection of a calculated model of the (10 R, 16 R)‐isomer of **4**. The calculated distance between HC 10 and HC 5’ of 3.2 Å is highlighted (color code: C gray, O red, N blue, H white).

To clarify the issue, whether the intriguing motif of a bicyclo‐[17.3.1]‐glycoside‐structure of the catabolite **4** would be a novel structural element in a natural tetrapyrrole, called for a systematic search in the literature. Checking specific organic compounds for novelty in appropriate databases is a routine operation nowadays.^23^ In our case, the question of the general topological relevance of a bridging‐pyranose moiety necessitated a substructure search for macrocycles related to **4** (bridging a pyranose ring), with particular emphasis on natural products. We utilized both Elsevier Reaxys^24^ and Chemical Abstracts Service (CAS) SciFinder.^25^

As the macrocycle, spanned by the bicyclo[17.3.1]glycoside‐structure **4**, contains 20 ring atoms, we searched for medium and large rings with 12 to 32 ring members (i.e., covering systems [9.3.1]–[28.3.1]) permitting carbon as well as heteroatom ring members in any combination, with any type of bonds outside the pyranose ring. Other features of **4** are the specific 1,5‐bridging of the pyranose ring, the lactone function within the macrocycle, and the presence of heterocyclic rings. Thus, for a necessary structural refinement of search results, stepwise restrictions were envisaged; first to those macrocycles with a properly positioned lactone function (Figure 6), further to compounds with one or more N‐atoms in the structure, and finally to those containing a generalized pyrrole‐type skeleton (four carbons and one nitrogen, any type of bond, any kind of position/annulation in the ring system). Thus, the number of hits was stepwise reduced from a starting total of 17 692 compound records in *SciFinder* (630 classified as natural products, see Supporting Information and Table S3) and 7 849 in *Reaxy*s (1 463 natural products) to lactones with one or more nitrogen atoms (413 in *SciFinder*, 321 in *Reaxys*), and finally to only 36 compounds (31 in *SciFinder*, 32 in *Reaxys*) containing an aza‐cyclopentyl (pyrrole‐type) skeleton.

**Figure 6:**
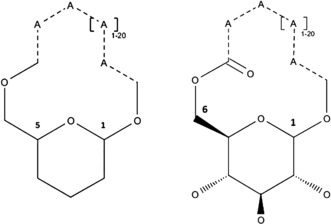
Left: Basic query structure for 1,5‐bridged pyranoses (A=any atom except hydrogen, dotted lines: any bond; queries and search results for other bridges are shown in the Supporting Information). Right: Refined query structure for 1,6‐bridged d‐glucopyranoses restricted to lactones (see the Supporting Information).

This first series of searches with increasing restrictions was repeated with macrocyles bridging d‐glucopyranose in the 1,6‐position (i.e., bicyclo[*n*.3.1]glycosides as in **4**; Figure 6) or other hexopyranoses: of the 36 aforementioned macrocycles containing a pyrrole‐type skeleton, 17 were 1,6‐bridged hexopyranoses, the majority (13) being d‐glucopyranoses (see Supporting Information, Tables S4 and S5). A designed glucopyranoside‐bridged porphyrin was identified,^26^ however, a natural product was not present. Thus, this search indicated that bicyclo[*n*.3.1]glycoside structures bridging five‐ring N‐heterocycles (related to those in **4**) are unprecedented. However, it furnished about 150 hits for bicyclo[*n*.3.1]glycosides as natural products with bridging by a lactone moiety in the range of organic linkers without N‐atoms. Interestingly, among these compounds inhibitors of cell growth27a or of high density induced apoptosis27b of human cancer cell lines, antibacterial,28a antifungal,28b–28d and antiviral compounds28e were found.

A further intriguing question raised by the discovery of the bicycloglycoside structure of *Ug*‐NCC‐53 (**4**) concerns how and at which stage of Chl‐breakdown is **4** generated. As a general rule, modifications reflected by the structures of NCCs are actually incorporated at the stage of FCCs by (mostly) cytosolic enzymes. NCCs with terminal sugar moieties at O 3^3^ have multiple precedence and appear to reflect corresponding glycosylations of their precursor FCCs. In contrast, attachment of a sugar moiety at the propionate group of Chl catabolites by esterification, has only been seen in the persistent “hypermodified” fluorescent Chl catabolites (*hm*FCCs)^4^, ^5^, ^6^ (Figure S1). Note that ester functions at the propionate side chain of FCCs stabilize them against their (acid‐induced) isomerization to the corresponding NCCs.6a, 14b, 29 As a consequence, *hm*FCCs accumulate in the blue luminescent peels of ripening bananas,6a,6c as well as in de‐greened leaves of the banana plant6b or of the peace lily, a tropical evergreen.6d A similarly esterified FCC was not observed here in the elm tree leaves. Furthermore, a bicycloglycosyl structure, as found in the NCC **4**, imposes stringent structural restraints on the structure of a phyllobilin. Probably, this would interfere with a regular acid‐induced isomerization of the FCC to a corresponding NCC.14a In the alternative case, in which the bridge motif of **4** was generated at the NCC stage, *Ug*‐NCC‐43 (**3**) would appear to be a potential biosynthetic precursor of **4** (Figure 4). As NCCs are localized in the vacuole,5b such a transformation would be required to take place in this confined environment. However, evidence for the metabolism of an NCC by a vacuolar enzyme is not available.5b All in all, the installation of the unique bicycloglycosyl motif of **4** remains puzzling.

Three major non‐fluorescent chlorophyll catabolites (NCCs) in naturally de‐greened leaves of the wych elm tree (*Ulmus glabra*) were found here to be glycosylated. One of these three 1‐formyl‐19‐oxophyllobilanes, named *Ug*‐NCC‐53 (**4**), carried an unprecedented bicycloglycosyl structure. This novel ring restrains the flexibility and imposes three‐dimensional features on the structure of the NCC **4** (Figure 4), which may be relevant in a still elusive role of this chlorophyll catabolite. This could involve activities related to those found in other bicyclo[*n*.3.1]glycosides (see above) or for example, in defence against bacterial or fungal infections.^30^ Clearly, the quest of the physiological relevance of NCCs or of other phyllobilins in senescent leaves and other plant organs5b, 6, 29 is a challenge to be pursued further.

## Experimental Section

**Spectroscopy**:All plant materials (yellow and green leaves) were collected in November 2010 from a wych elm tree (*Ulmus glabra*) at the main campus of the University of Innsbruck, and were analyzed freshly, or stored at −80 °C for further use.

*UV/Vis*: Hitachi U‐3000, *λ*_max_ [nm] (log ɛ). ^*1*^ *H‐ and* ^*13*^ *C NMR*: Bruker 600 MHz Avance II+; residual solvent peaks (CD_2_HOD: *δ*_H_=3.31 ppm; ^13^CD_3_OD: *δ*_C_=49.0 ppm).^31^ *Mass spectrometry*: Finnigan MAT 95 (MS), electrospray ionization (ESI) source, positive ion mode, 1.4 kV spray voltage; Finnigan LCQ Classic (LC‐MS), ESI source, positive ion mode, 4.5 kV spray voltage; *m*/*z* (relative intensity). *HPLC*: An extract from a freshly picked leaf (area of about 15 cm^2^) of a wych elm tree was analyzed by HPLC (detection at 320 nm, see Supporting Information for further experimental. details, and Figure S2 A, B).

**Isolation and structure elucidation of Ug‐NCCs**: From 115 g of senescent leaves (wet weight), 7.1 mg (8.4 μmol) of *Ug*‐NCC‐27 (**2**), 12.2 mg (15.1 μmol) of *Ug*‐NCC‐43 (**3**), and 1.2 mg (1.5 μmol) of *Ug*‐NCC‐53 (**4**) were isolated, which showed the following spectro‐analytical data (see Supporting Information for further details). *Ug*‐NCC‐27 (**2**): UV/Vis (MeOH, *c*=4.1×10^−5^ m): *λ*_max_ (*ɛ*_rel_)=240 (1.0), 311 nm (1.0); LC‐MS (ESI^+^): *m*/*z* (%): 879.36 (59, [*M*+K]^+^); 843.39 (14.20), 842.39 (39), 841.42 (100, [*M*+H]^+^, C_41_H_53_N_4_O_15_^+^); *Ug‐*NCC‐43 (**3**): UV/Vis (MeOH, *c*=6.82×10^−5^ m): *λ*_max_ (*ɛ*_rel_)=248 (1.00), 313 nm (0.98); LC‐MS (ESI^+^): *m*/*z* (%): 845.43 (48, [*M*+K]^+^); 809.48 (13), 808.48 (55), 807.48 (100, [*M*+H]^+^, C_41_H_51_N_4_O_13_^+^); *Ug*‐NCC‐53 (**4**): UV/Vis (MeOH, *c*=3.2×10^−5^ m): *λ*_max_ (*ɛ*_rel_)=242 (sh, 4.39), 312 nm (4.33); MS (ESI^+^): *m*/*z* (%): 827.20 (50, [*M*+K]^+^); 811.27 (78, [*M*+Na]^+^), 791.20 (27), 790.20 (75), 789.20 (100, [*M*+H]^+^, C_41_H_49_N_4_O_12_^+^.

**Molecular modeling**: NCC **4** and the C 10‐ and C 16‐epimeric versions of it were constructed using MOE 2013.08 (Chemical Computing Group Inc., Montreal, QC, Canada). Partial charges were obtained by using the AM1‐BCC semi‐empirical method,^32^ as implemented in the antechamber tool of the AmberTools 13 package.^33^ All species were hydrated in octahedral periodic boxes of approximately 3000 TIP3P water molecules.^34^ Bond, angle, and torsion potentials were modelled using the generalized AMBER force field (GAFF) version 1.5.^35^ All systems were equilibrated for 100 ns using a van der Waals cut‐off of 8.0 Å, particle mesh Ewald electrostatics,^36^ a pressure of 1.0 atm by Berendsen weak coupling^37^ and a temperature maintained at 300 K by a Langevin thermostat.^38^ Shake^39^ was enabled on all bonds to hydrogen to allow for a simulation time step of 0.2 fs. Subsequently, 200 ns of sampling were obtained for each system using the GPU implementation of pmemd.^40^ One nanosecond running averages of the distances (H_3_C 8^5^)H 1,2,3–H 5′, HC 10–H 5′, HC 10–H_A/B_C 12^1^, and HC 10–H_A/B_C 12^2^ were computed for the duration of the simulation using “ptraj” from the AmberTools 13 package and are given in the Supporting Information (Figure S11–14).

**Literature search**: Substructure searches were executed in CAS SciFinder^24^ (non‐Java structure editor, query structures saved in cxf format; “Explore Substances” – “Chemical Structure” – “Substructure”, no restrictions concerning salts, mixtures, isotopes etc.) and Elsevier Reayxs^25^ (ChemAxon Marvin Sketch 6.0.6 and earlier versions, query structures saved in mrv format; “Substances,Names,Formulas” – “Structure” – “Substructure on all atoms”, no restrictions concerning salts, mixtures, isotopes etc.): final search for the data given here was performed on Jan 10th, 2016 (Reayxs: Version 2.20770.1, last update Jan 7th, 2016; SciFinder: Version December 2015), preceded by earlier searches in April & June 2015, and in June 2014, first exploratory searches in April 2014. In SciFinder, the literature was limited to the CAPLUS database. For details about search strategies, queries, and results see the Supporting Information.

## Supporting information

As a service to our authors and readers, this journal provides supporting information supplied by the authors. Such materials are peer reviewed and may be re‐organized for online delivery, but are not copy‐edited or typeset. Technical support issues arising from supporting information (other than missing files) should be addressed to the authors.

Supplementary
