# Supplementary material for: Chlorophyll Catabolites in Fall Leaves of the Wych Elm Tree Present a Novel Glycosylation Motif
Source: Chemistry. 2016 Jun 6;22(28):9498–503. doi: 10.1002/chem.201601739 (PMC5089558; doi:10.1002/chem.201601739)
Supplement: Supplementary file 1 — Supplementary [file CHEM-22-9498-s001.pdf]

CHEMISTRY
A European Journal

Supporting Information

Chlorophyll Catabolites in Fall Leaves of the Wych Elm Tree Present a Novel Glycosylation Motif

Mathias Scherl,[a] Thomas Müller,[a] Christoph R. Kreutz,[a] Roland G. Huber,[c, d] Engelbert Zass,*[b] Klaus R. Liedl,*[c] and Bernhard Kräutler*[a]

chem_201601739_sm_miscellaneous_information.pdf

Chlorophyll Catabolites in Fall Leaves of the Wych Elm Tree Present a Novel Glycosylation Motif

Supporting Information

Mathias Scherl[a,b], Thomas Müller[a,b], Christoph R. Kreutz[a,b], Roland G. Huber[b,c], Engelbert Zass[d],* Klaus R. Liedl[b,c*] and Bernhard Kräutler[a,b*]

a Institute of Organic Chemistry, University of Innsbruck

Innrain 80/82, A-6020 Innsbruck (Austria)

E-mail: bernhard.kraeutler@uibk.ac.at

b Center of Molecular Biosciences, University of Innsbruck

Innrain 80/82, A-6020 Innsbruck (Austria)

c Institute of General, Inorganic & Theoretical Chemistry, University of Innsbruck

Innrain 80/82, A-6020 Innsbruck (Austria)

E-mail: Klaus.Liedl@uibk.ac.at

d Laboratory of Organic Chemistry, ETH Zürich

Vladimir-Prelog-Weg 3, CH-8093 Zürich (Switzerland)

E-mail: zass@retired.ethz.ch

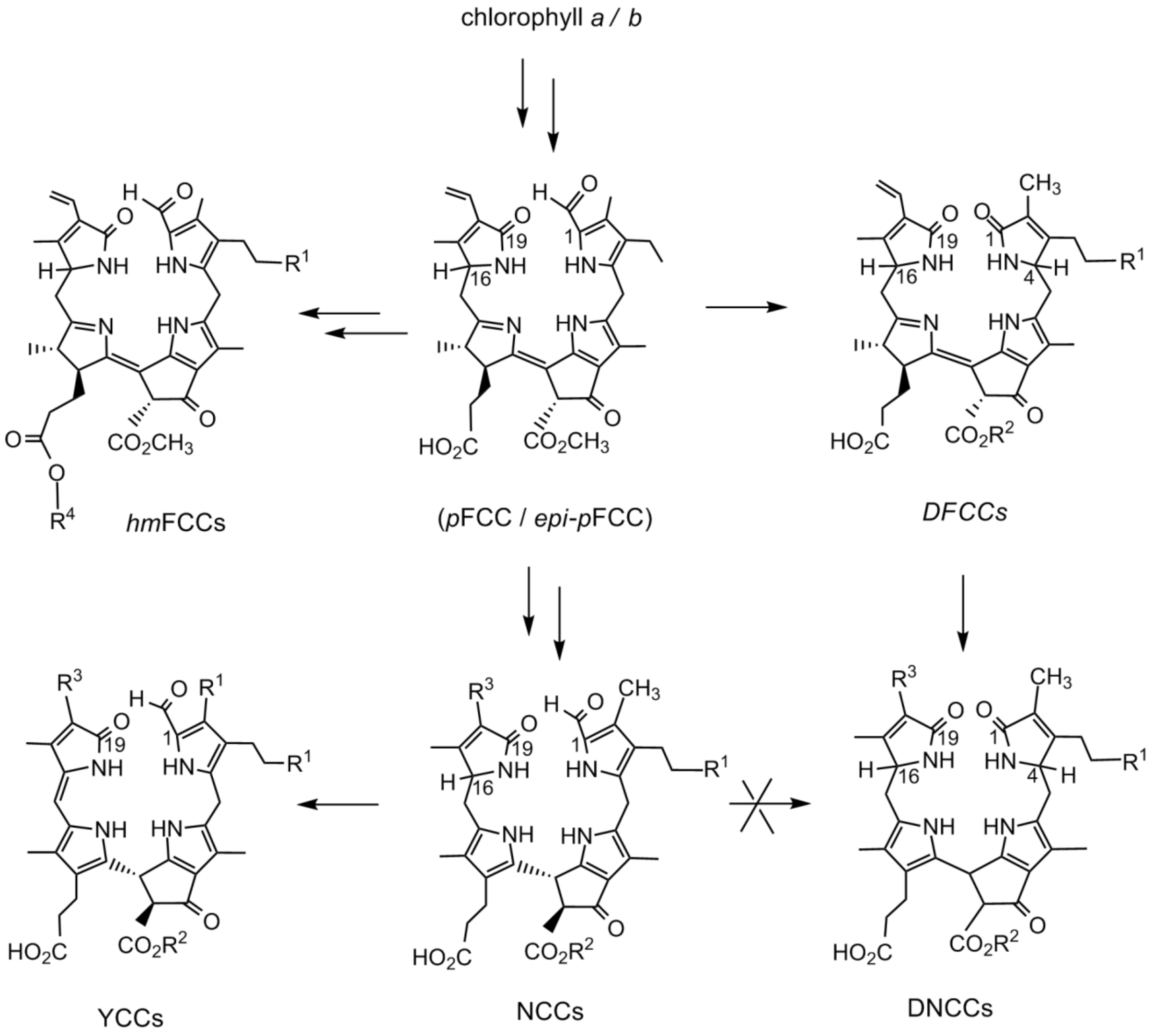

Figure S1. Structural outline of Chl-breakdown in senescent leaves. In the early steps chlorophylls *a* & *b* are degraded to the ´primary` fluorescent Chl-catabolite (*p*FCC) or to its 16-epimer (*epi*-*p*FCC). *p*FCCs and *epi*-*p*FCCs undergo modifications, followed by isomerization to several ‘nonfluorescent’ Chl-catabolites (NCCs), or they are esterified to ‘hypermodified’ FCCs (*hm*FCCs). Enzymatic deformylation of FCCs may, alternatively, provide dioxobiline-type FCCs (DFCCs), which isomerize to dioxobiline-type NCCs (DNCCs). Yellow Chl-catabolites (YCCs) were found in some senescent, yellow leaves as oxidation products of NCCs. [1]

General.

Plant material. Green and yellow leaves were collected in August or November 2010 respectively, from a Wych elm tree (*Ulmus glabra*) at the main campus of the University of Innsbruck, and were analyzed freshly or stored at -80°C for further use.

Chemicals. Commercial solvents (reagent-grade) were redistilled before use for extractions. HPLC-Grade methanol (MeOH), n-hexane and acetonitrile (ACN) were purchased from *Merck* (Darmstadt), *Acros Organics* and *Sigma Aldrich*. Potassium dihydrogen phosphate *puriss. p.a.*, potassium phosphate dibasic-anhydrous *puriss. p.a.* and ammonium acetate *puriss. p.a.* were from *Fluka* (CH-Buchs). *Sep-Pak-C18* Cartridges (5 g and 1 g sizes) were from *Waters Associates*. The *pH* values were measured with a WTW *Sentix 21* electrode connected to a *WTW pH535* digital *pH*-meter.

HPLC: Dionex Summit HPLC system with manual sampler, *P680* pump, online degasser and diode array detector, 1 ml or 20 µl injection loop. Data were collected and processed with Chromeleon V6.50. *Dionex Ultimate 3000* HPLC system, ultimate *3000 pump*, ultimate 3000 diode array detector and *RF2000* fluorescence detector, 20 µl or 100 µl injection loop. a) Anal. *HPLC: Phenomenex HyperClone ODS* 5 µm 250 x 4.6 mm i.d. column at 20° protected with a *Phenomenex ODS* 4 mm x 3 mm i.d. pre-column was used with a flow rate 0.5 ml min^{-1}. Solvent *A*: 50 mM aq. potassium phosphate (*pH* 7.0), solvent *B*: MeOH; solvent composition: (*A*/*B*) 0 – 5 min: 80/20; 5-55 min: 80/20 to 30/70; 55 – 60 min: 30/70 to 0/100; 60 – 70 min: 0/100; 70-75 min: 0/100 to 80/20. *b)* Prep. HPLC*: Phenomenex HyperClone ODS* 5 µm 250 x 21.2 mm i.d. column at 20° protected with a *Phenomenex ODS* 10 mm x 5 mm i.d. pre-column was used with a flow rate 5 ml min^{-1}. Solvent *A*: 50 mM aq. potassium phosphate (*pH* 7.0), solvent *B*: MeOH. *LC-MS:* HPLC: *Dionex Ultimate* HPLC system with manual sampler, helium degasser and diode array detector. 20 µl injection loop. *Phenomenex HyperClone ODS* 5 µm 250

x 4.6 mm i.d. column at 20° protected with a *Phenomenex ODS* 4 mm x 3 mm i.d. pre-column was used with a flow rate 0.5 ml min^{-1}. Solvent *C*: 10 mM ammonium acetate (*pH* 7.0), solvent *B*: MeOH; solvent composition (*C*/*B*) 0 – 5 min: 80/20; 5-55 min: 80/20 to 30/70; 55 – 60 min: 30/70 to 0/100; 60 – 70 min: 0/100; 70 - 75 min: 0/100 to 80/20. Data were collected and processed with Chromeleon V6.50.

Spectroscopy. UV/Vis: *Hitachi U-3000* spectrophotometer; λ_{max} (nm)/(logε). CD: JASCO J715 λ_{max} and λ_{min} (nm)/$\Delta\varepsilon$. *^{1}H- and ^{13}C-NMR:* Bruker 600 MHz Avance II+ (δ(C^1HD_2OH) = 3.31 ppm, and δ ($^{13}CD_3OD$) = 49.0 ppm) [2,3], ^{13}C-signal assignment from HSQC and HMBC experiments [4]. Mass spectrometry: *Finnigan MAT 95*, electrospray ionization (ESI) source, positive ion-mode, 1.4 kV spray voltage; MS and MS/MS: *Finnigan LCQ Classic*, ESI source, positive ion-mode, 4.5 kV spray voltage (rel. abundance) [Müller et al, 2014]. Bruker Ultraflex MALDI TOF mass spectrometer equipped with a smartbeam2 Nd:YAG-laser (effective spot size of 35 µm). The laser power was adjusted and kept at 35% (manufacture´s arbitrary units); 2,5-dihydroxybenzoic acid was used as matrix; PPG 1000 as internal standard.

Analysis of Chl-catabolites by HPLC. A freshly picked Wych Elm tree leaf (with the area of about 15 cm^2) was ground in a mortar and extracted with 2 ml of MeOH. The resulting suspension was centrifuged 5 minutes at 13,000 rpm. The methanolic supernatant was diluted with 50 mM aq. potassium phosphate (*pH* 7.0) 50:50 (*v/v*) and centrifuged for 5 minutes at 13,000 rpm. From the supernatant 20 µl were injected into the anal. HPLC-system (detection at 320 nm, see Figure S2A).

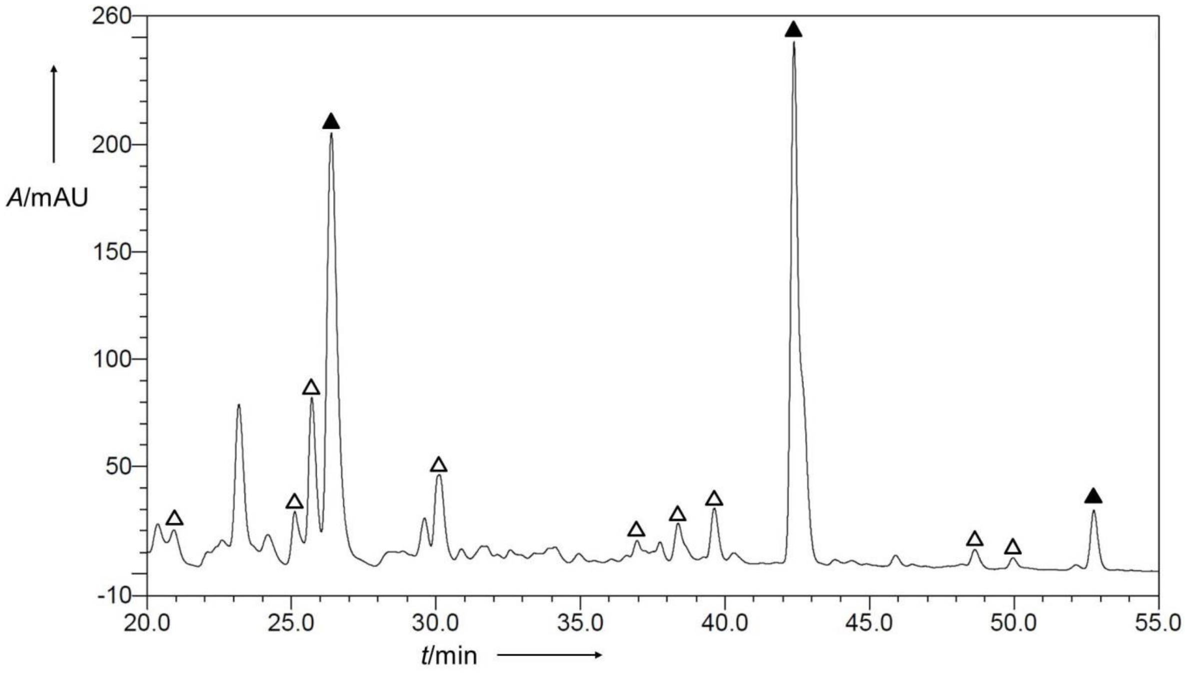

Figure S2A. HPLC analysis of an extract of senescent leaves of *Ulmus glabra* (10 mM ammonium acetate buffer, detection at 320 nm), NCCs **2, 3, 4** are denoted with filled triangle (▲), other fractions classified as NCCs are highlighted with an open triangle (△).

Quantification of Chl in green and senescent Elm tree leaves by UV/Vis Spectroscopy.

Chl a and b in green leaves. A total area of 9 cm^2 was cut out of a green *Ulmus glabra* leaf. The leaf was frozen in liquid N_2, pulverized in a mortar, and extracted with MeOH. The slurry was filtered through a sintered glass filter, and the residue was ground in a mortar and extracted with MeOH. The procedure was repeated until the residue was colorless. The extracts were combined and diluted with MeOH to 100.00 ml in a volumetric flask. After filtration using a Sartorius filter the extracts were analyzed by UV/Vis spectrometry. In green *Ulmus glabra* leaves 51.1 ± 3.7 µg cm^{-2} (56.9 ± 4.2 nmol cm^{-2}) of chlorophyll a and b were found (n=4), the data analysis was based on ref. [5].

Chl a and b in senescent leaves: A total area of 141 cm^2 (second and third measurement: 81 cm^2) was cut out of several senescent *Ulmus glabra* leaves. The extraction and the UV/Vis analysis

were performed as described above. Yellow *Ulmus glabra* leaves were found to contain 0.5 ± 0.3 μg cm^{-2} (0.5 ± 0.1 nmol cm^{-2}) of residual chlorophyll a and b (n=3).

Quantification of nonfluorescent Chl-catabolites* (NCCs) *in senescent leaves by anal. HPLC. *NCCs in senescent (yellow) leaves:* The quantification of the *Ug*-NCCs was accomplished by anal. HPLC. A sample of *Cj*-NCC-1 [6] from *Cercidiphyllum japonicum* was used to prepare a standard soln. (calculated with ε^{320} = 17,000 as described). Senescent *Ulmus glabra* leaves were found to contain 16.8 ± 2.2 μg cm^{-2} (20.0 ± 2.6 nmol cm^{-2}) of *Ug*-NCC-27, 11.5 ± 1.5 μg cm^{-2} (14.2 ± 1.8 nmol·cm^{-2}) of *Ug*-NCC-43 and 1.7 ± 0.4 μg cm^{-2} (1.9 ± 0.4 nmol cm^{-2}) of *Ug*-NCC-53. These values indicate, in a yellow *Ulmus glabra* leaf, a total conversion of chlorophyll a and b to NCCs of about 63 %.

Collection, isolation and structure elucidation of NCCs.

Senescent (yellow) Whych elm tree leaves (60 g, wet weight) were frozen in liquid nitrogen, crushed into small pieces with a 300 W blender and pulverized portion wise in liquid nitrogen and in a mortar. Following extraction with 120 ml MeOH, the suspension was filtered through a Buchner funnel. The extraction was repeated two times using 120 ml of MeOH each time. The combined methanolic extracts (370 ml) were filtered through a paper filter and concentrated to 50 ml using a rotary evaporator. After washing the extract with n-hexane (50 ml), the methanolic phase was diluted with 50 mM aq. potassium phosphate (*pH* 7.0) / MeOH 80:20 (*v*/*v*). The solution was then applied to a Sepak-cartridge (5g), washed with 20 ml of 50 mM potassium phosphate (*pH* 7.0) / MeOH 80:20 (*v*/*v*) and eluted with MeOH. The NCC-enriched methanolic solution was concentrated using a rotary evaporator, was dried in vacuo to yield an NCC-enriched raw product (dry-weight: 0.359 g), which was stored at -80°C for further purification.

The preparation of raw product was repeated with a second batch of senescent leaves (55 g, wet weight) giving a raw product sample of 0.244 g.

The combined raw product (0.623 g) was dissolved in 1.5 ml MeOH and 1.5 ml aq. potassium phosphate buffer soln. (50 mM; *pH* 7.0) using an ultra-sonic bath. Following centrifugation at 13,000 rpm (3 min) the suspension was filtered through a Sartorius filter (Chromafil CA-20 / 25 S, 0.20 µm cellulose acetate). The eluate was divided in five aliquots and to be applied to prep. HPLC; injection volume: 1.5 ml; flow rate: 5 ml min^{-1}; solvent *A*: 50 mM aq. potassium phosphate (*pH* = 7.0); solvent *B*: MeOH; solvent composition: (*A*/*B*): 0-120 min: 80/20 to 65/35; 120-180 min: 65/35 to 0/100. Five consecutive preparative HPLC runs were performed and fractions were collected containing *Ug*-NCC-27 (between 47.0 and 52.5 min), *Ug*-NCC-43 (between 85.8 and 89.8 min) and *Ug*-NCC-53 (between 103.8 and 107 min). The three fractions were collected, applied to a Sepak-cartridge (1g), and washed with 20 ml of water. They were then eluted with a minimum amount of MeOH. The solvents were removed using a rotary evaporator and by drying under high vacuum.

The sample of *Ug*-NCC-27 (**2**), so obtained, was further purified by preparative HPLC (injection volume: 1.0 ml; flow rate: 5 ml min^{-1}; solvent composition *A*/*B* 0-220 min: 92/8 to 65/35), desalting using a 1 g Sepak-cartridge (see above) and drying under high vacuum, to give 7.1 mg (8.4 µmol) of analytically pure **2**.

Likewise, the sample of *Ug*-NCC-43 (**3**), so obtained, was further purified by preparative HPLC (injection volume: 1.0 ml; flow rate: 5 ml.min^{-1}; solvent composition: *A*/*B* 0-180: 80/20 to 65/35), desalting (see above) and drying under high vacuum, to yield 12.2 mg (15.1 µmol) of analytically pure **3**.

For further purification the sample of *Ug*-NCC-53 (**4**) was similarly purified by preparative HPLC (injection volume: 1.0 ml; flow rate: 5 ml min^{-1}; solvent composition *A*/*B* 0-150 min:

80/20 to 40/60), de salted (see above) and dried under high vacuum. Thereby 4.3 mg of the catabolite **4** were obtained. Final purification was achieved in smaller batches using the analytical HPLC system (injection volume: 0.1 ml; flow rate: 0.5 ml min^{-1}; solvent *C*: 10 mM ammonium acetate (*pH* 7.0); solvent *D*: ACN; solvent composition *C*/*D* 0-5 min: 80/20; 5 - 55 min: 80/20 to 60/40; 55 - 63 min: 60/40 to 5/95; 63 - 73 min: 5/95; 73-80 min: 5/95 to 80/20). After desalting (see above) and drying under high vacuum 1.2 mg (1.5 µmol) of analytically pure *Ug*-NCC-53 (**4**) were obtained.

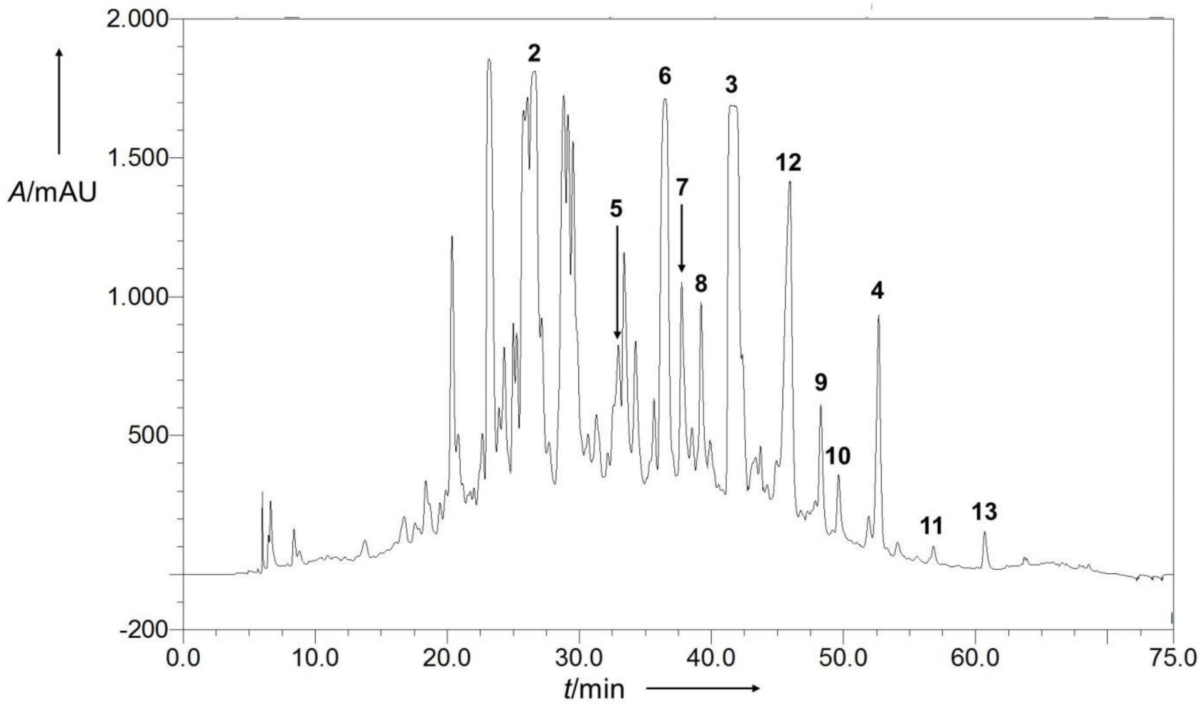

Figure S2B. HPLC analysis of an extract of senescent leaves of *Ulmus glabra* (10 mM ammonium acetate buffer, detection at 320 nm) obtained from the bulk isolation experiment (see collection, isolation and structure elucidation of *Ug*-NCCs). NCC fractions are numbered **2 -11**, two YCCs are numbered **12** and **13** (see below for further data).

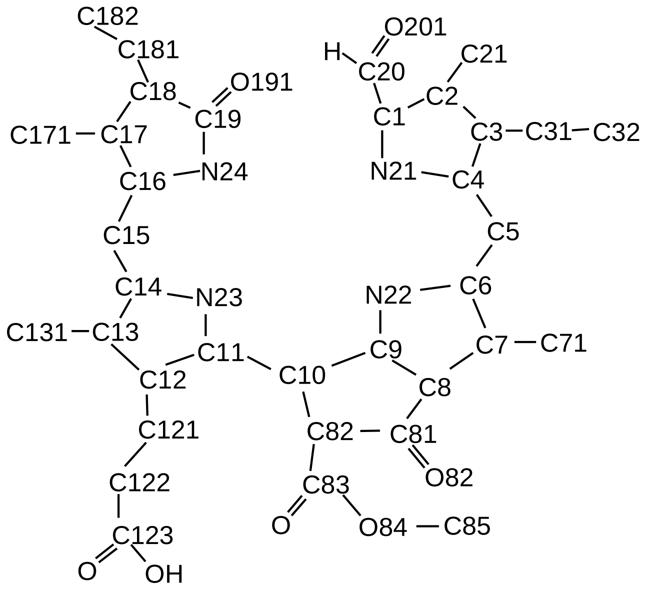

Figure S3. Atom numbering used for the phyllobilin skeleton. [7]

Spectroanalytical data: *Ug*-NCC-27 (2, see Figure S6 for a formula). UV/Vis (MeOH, c = 4.1 * 10^{-5} M): λ_{max} (rel ε) = 240 (1.0), 311 (1.0) nm. CD (MeOH, c= 4.1 * 10^{-5} M); λ_{max} / nm ($\Delta\varepsilon$): 225 (14), 282 (-5), 314 (5). ^{1}H NMR (600 MHz, 20°, in CD_3OD): δ = 1.92 (*s*, 3H, H_3C-13^1); 2.04 (*s*, 3H, H_3C-17^1); 2.10 (*s*, 3H, H_3C-7^1); 2.24 (*s*, 3H, H_3C-2^1); 2.28 – 2.33 (*m*, 2H, H_2C-12^2); 2.49 (*dd*, 1H, *J* = 8.7 / 14.5, H_AC-15); 2.59 - 2.65 (*m*, 1H, H_AC-12^1); 2.66 (*triplettoid*, 2H, *J* ≈ 7.2, H_2C-3^1); 2.69 – 2.77 (*m*, 1H, H_BC-12^1); 2.90 (*dd*, 1H, *J* = 4.3 / 14.5, H_BC-15); 3.17 (*dd*, 1H, *J* = 7.9 / 9.1, HC-2´); 3.25 – 3.27 (*m*, (HC-4´), (HC-5´), 2H); 3.34 – 3.43 (*m*, (H_AC-3^2), (HC-3´), 2H); 3.61 – 3.66 (*m*, (H_AC-18^2), (H_AC-6´), 2H); 3.69 (*dd*, 1H, *J* = 6.5 / 11.3, H_BC-18^2); 3.75 ((*m*, H_BC-3^2), superimposed by 3.75 (*s*, H_3C-8^5), 4H); 3.82 – 3.87 (*m*, H_BC-6´); 3.98 (*d*, 1H, *J* = 17.0, H_AC-5); 4.04 (*dd*, 1H, *J* = 4.3 / 8.7, HC-16); 4.06 (*d*, 1H, *J* = 17.0, H_BC-5); 4.19 (*d*, 1H, *J* = 7.6 (HC-1´); 4.56 (*dd*, 1H, *J* = 5.3 / 6.3, HC-18^1); 4.88 (*s*, 1H, HC-10); 9.32 (*s*, HC-1^1).^{13}C-NMR (150 MHz, CD_3OD, 20°): δ [ppm] = 8.5 (2^1), 9.1 (13^1), 9.2 (7^1), 12.3 (17^1), 21.8 (12^1), 23.6 (5), 24.9

(3^1), 29.5 (15), 36.9 (10), 39.1 (12^2), 52.5 (8^5), 62.3 (6´), 62.3 (16), 65,6 (18^2), 67.8 (8^2), 68.3 (18^1), 70.1 (3^2), 71.2 (4´), 74.8 (2´), 77.5 (5´), 77.7 (3´), 103.9 (1´), 112.0 (7), 115.0 (13), 120.2 (12), 124.0 (14), 124.2 (11), 120.4 (3), 125.8 (8), 128.9 (1), 131.4 (18), 133.9 (6), 135.2 (2), 139.1 (4), 158.7 (17), 161.0 (9), 171.3 (8^3), 174.8 (19), 177.3 (1^1), 180.8 (12^3), 191.5 (8^1). LC-MS (ESI): *m/z* (%) = 879.36 (59, $[M+K]^+$); 863.34 (11, $[M+Na]^+$); 843.39 (14.20), 842.39 (39), 841.42 (100, $[M+H]^+$; $C_{41}H_{53}N_4O_{15}^+$); 809.40 (6, $[M+H-CH_3OH]^+$); 684.25 (28, [M+H-$C_7H_{11}NO_3$ (ring D)]$^+$); 679.27 (11; [M+H-$C_6H_{10}O_5$ (sugar moiety)]$^+$).

***Ug*-NCC-43 (3,** see Figure S6 for a formula). UV/Vis (MeOH, c = 6.82 $^*10^{-5}$ M): λ_{max} (rel ε) = 248 (1.00), 313 (0.98) nm. CD (MeOH, c = 6.82 * 10^{-5} M); λ_{max} / nm ($\Delta\varepsilon$): 225 (17), 258sh (-5), 282 (-12), 316 (3). ^{1}H-NMR (600 MHz, CD_3OD, 20°): δ [ppm] = 1.92 (*s*, 3H, H_3C-13^1); 1.98 (*s*, 3H, H_3C-17^1); 2.12 (*s*, 3H, H_3C-7^1); 2.22 (*s*, 3H, H_3C-2^1); 2.32 (*triplettoid*, 2H, $J \approx 7.2$ H_2C-12^2); 2.42 (*dd*, 1H, J = 9.4 / 14.4, H_AC-15); 2.60-2.68 (*m*, (H_2C-3^1), (H_AC-12^1), 3H); 2.68-2.79 (*m*, 1H, H_BC -12^1); 2.88 (*dd*, 1H, J = 4.8 / 14.4, H_BC-15); 3.17 (*dd*, 1H, J = 7.9 / 9.1, HC-2´); 3.24-3.27 (*m*, (HC-4´), (HC-5´), 2H); 3.33-3.44 (*m*, (HC-3´), (H_AC-3^2), 2H); 3.64 (*dd*, 1H, J = 5.0 / 11.9, H_AC-6´); 3.76 ((*m*, H_BC-3^2) superimposed by 3.76 (*s*, H_3C-8^5), 4H); 3.78-3.90 (*m*, 1H, H_BC-6´); 3.95 (*d*, 1H, J = 17.1, H_AC-5); 4.01 (*dd*, 1H, J = 4.8 / 9.4, HC-16); 4.04 (*d*, 1H, J = 17.1, H_BC-5); 4.19 (*d*, 1H, J = 7.9, HC-1´); 4.88 (*singlettoid*, 1H, HC-10); 5.35 (*dd*, 1H, J = 2.2 / 11.7, H_AC-18^2); 6.10 (*dd*, 1H, J = 2.2 / 17.8, H_BC-18^2); 6.44 (*dd*, 1H, J = 11.7 / 17.8 HC-18^1); 9.29 (*s*, 1H, HC-1^1). ^{13}C-NMR (150 MHz, CD_3OD, 20°), δ [ppm] = 8.6 (2^1); 9.2 (7^1); 9.2 (13^1); 12.3 (17^1); 21.7 (12^1); 23.6 (5); 24.8 (3^1); 30.1 (15); 36.9 (10); 38.7 (12^2); 52.5 (8^5); 61.5 (16); 62.3 (6´); 67.7 (8^2); 70.1 (3^2); 71.1 (4´); 74.8 (2´); 77.7 (3´); 77.5 (5´); 103.7 (1´); 112.2 (7); 115.2 (13); 118.7 (18^2); 120.1 (12); 120.3 (3); 124.2 (11); 124.4 (14); 125.7 (8); 126.7 (18^1); 128.3 (18); 128.7 (1); 133.9 (6); 135.2 (2); 139.0 (4); 156.5 (17); 160.9 (9); 171.2 (8^3); 174.3 (19); 177.1 (1^1); 180.0 (12^3). LC-MS (ESI): *m/z* (%) = 883.29 (4, $[M-H+2K]^+$), 867.41 (6, $[M-H+K+Na]^+$); 845.43 (48

[M+K]$^+$); 829.42 (17, [M+Na]$^+$); 809.48 (13), 808.48 (55), 807.48 (100, [M+H]$^+$; $C_{41}H_{51}N_4O_{13}^+$); 775.43 (15, [M+H-CH_3OH]$^+$), 684.35(8; [M+H-C_7H_9NO (ring A)]$^+$), 645.30 (11; [M+H-$C_6H_{10}O_5$ (sugar moiety)]$^+$).

***Ug*-NCC-53 (4**, see Figure S6 for a formula). UV/Vis (MeOH, *c* =3.2 * 10^{-5} M) λ_{max} (log ε) = 242 sh (4.39), 312 (4.33) nm. CD (MeOH, *c*= 3.4 * 10^{-5} M) λ_{max} / nm ($\Delta\varepsilon$): 225 (31), 256 sh (-4), 283 (-21), 315 (4) (see Figure S4). ^{1}H-NMR (600 MHz, CD_3OD, 10°): δ [ppm] = 1.89 (*s*, 3H, H_3C-13^1); 2.01 (*s*, 3H, H_3C-17^1); 2.13 (*s*, 3H, H_3C-7^1); 2.27 (*s*, 3H, H_3C-2^1); 2.31-2.39 (*m*, 2H, H_2C-12^2); 2.40-2.47 (*m*, 1H, H_AC-12^1); 2.50 (*dd*, 1H, *J* = 8.6 / 14.6, H_AC-15); 2.60-2.68 (*m*, 1H, H_BC-12^1); 2.70 (*t*, 2H, *J* = 6.7, H_2C-3^1); 2.89 (*dd*, 1H, *J* =5.1 / 14.6, H_BC-15); 3.13 (*dd*, 1H, *J* = 7.9 / 9.1, HC-2´); 3.25 (*triplettoid*, 1H, *J* ≈ 9.5, HC-4´); 3.35 (*triplettoid*, 1H, *J* ≈ 9.5, HC-3´); 3.45-3.53 (*m*, (*J* = 2.3 / 6.7, HC-5´), (H_AC-3^2), 2H); 3.63-3.69 (*m*, 1H, H_BC-3^2); 3.76 (*s*, 3H, H_3C-8^5); 3.78 (*d*, 1H, *J* = 3.1, HC-8^2); 3.91 (*d*, 1H, *J* = 17.0, H_AC-5); 3.97 (*d*, 1H, *J* = 17.0, H_BC-5); 3.97 (*dd*, 1H, *J* = 6.7 / 12.3, H_AC-6´); 4.09 (*dd*, 1H, *J* = 5.1 / 8.6, HC-16); 4.23 (*d*, 1H, *J* = 7.9, HC-1´); 4.56 (*duplettoid*, *J* = 2.3 / 12.3, H_BC-6´); 4.88 (buried under water signal, HC-10); 5.35 (*dd*, 1H, *J* = 2.2 / 11.7, H_AC-18^2); 6.10 (*dd*, 1H, *J* = 2.2 / 17.7, H_BC-18^2); 6.45 (*dd*, 1H, *J* = 11.7 / 17.7, HC-18^1); 9.32 (*s*, 1H, HC-20).^{13}C-NMR (150 MHz, CD_3OD, 10 °C): δ [ppm] = 8.8 (2^1); 9.0 (7^1); 9.0 (13^1); 12.5 (17^1); 19.8 (12^1); 23.6 (5); 25.4 (3^1); 30.1 (15); 36.1 (12^2); 38.1 (10); 52.7 (8^5); 61.5 (16); 64.7 (6´); 68.1 (8^2); 71.6 (3^2); 71.6 (4´); 74.8 (2´); 75.2 (5´); 77.8 (3´); 104.6 (1´); 112.5 (7); 115.5 (13); 119.0 (18^2); 119.8 (12); 121.3 (3); 123.9 (14); 124.5 (11); 125.8 (8) 126.8 (18^1); 128.6 (18); 129.2 (1); 134.2 (6); 135.5 (2); 138.3 (4); 156.6 (17); 160.9 (9); 171.6 (8^3); 174.4 (12^3); 174.6 (19); 177.5 (20). MS (ESI): *m/z* (%) = 827.20 (50,[M+K]$^+$); 811.27 (78, [M+Na]$^+$), 791.20 (27), 790.20 (75),

789.20 (100, $[M+H]^+$, $C_{41}H_{49}N_4O_{12}{}^+$); 757.20 (10, $[M+H\text{-}CH_3OH]^+$); 666.27 (30, [M+H-C_7H_9NO (ring D)]$^+$); 634.27 (3, [M+H-ring D - $CH_3OH]^+$). MS/MS of $[M+H^+]$ at *m/z* 789.2: *m/z* (%) = 771.13 (2, $[M+H\text{-}H_2O]^+$; 757.13 (100, $[M+H - CH_3OH]^+$); 739.13 (2, $[M+H\text{-}MeOH\text{-}H_2O]^+$; 666.13 (24, [M+H-ring D]$^+$); 634.13 (2, M+H-ring D –$CH_3OH]^+$).

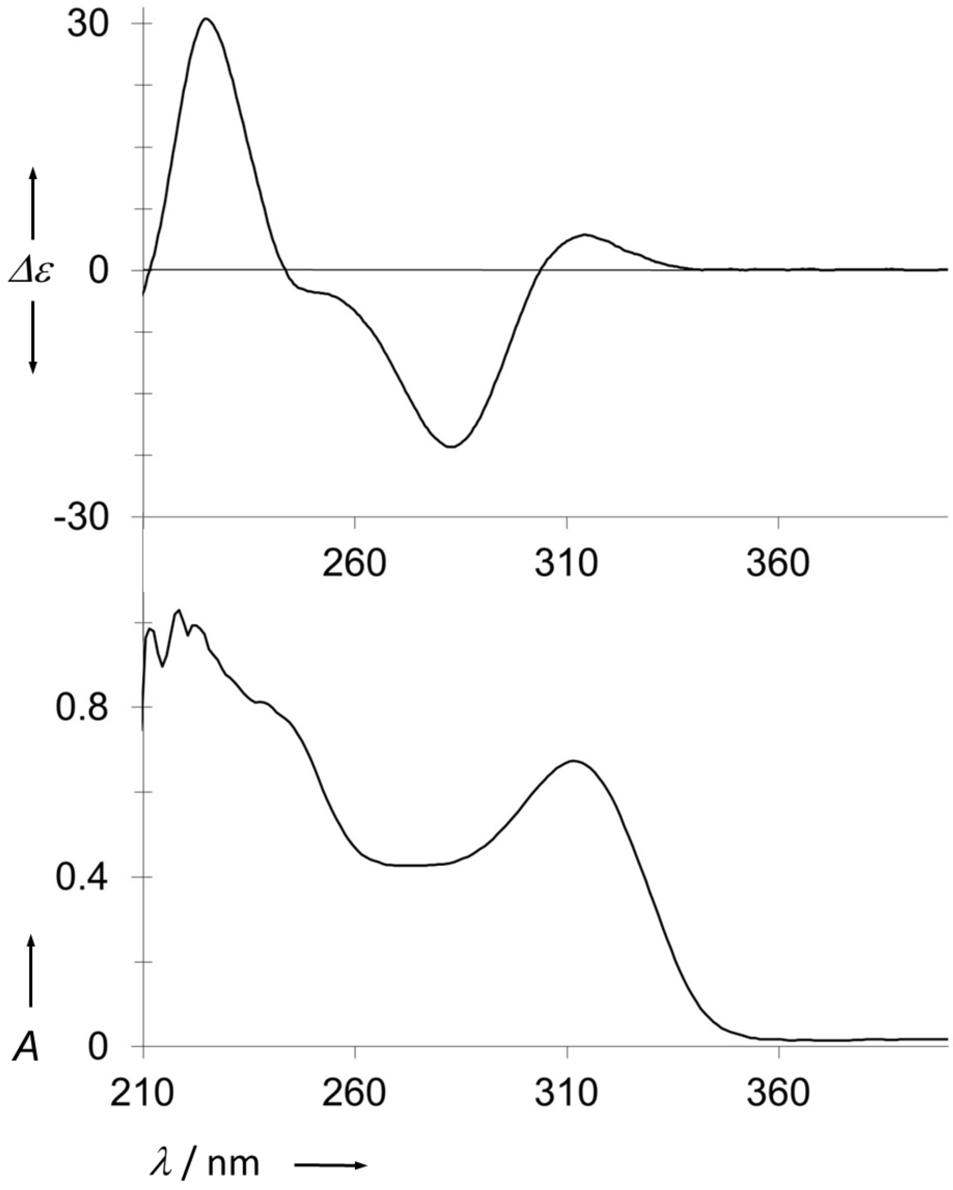

Figure S4. CD spectrum (top) and UV/Vis spectrum (bottom) of *Ug*-NCC-53 (**4**) in MeOH.

Table S1. Assignment of ^{1}H-signals from 600 MHz ^{1}H-NMR spectra of *Ug*-NCC-43 (**3**) and *Ug*-NCC-53 (**4**) in CD_3OD; ^{13}C assignment from correlations with HSQC and HMBC spectra.

**assignment**	*Ug*-NCC-43 (**3**) (30.2 mM)		*Ug*-NCC-53 (**4**) (3.0 mM)	
	δ(^{1}H)/ppm	δ(^{13}C)/ppm	δ(^{1}H)/ppm	δ(^{13}C)/ppm
**C-16**	4.01	61.5	4.09	61.5
**C-17**		156.5		156.6
**C-17^{1}**	1.98	12.3	2.01	12.5
**C-18**		128.3		128.6
**C-18^{1}**	6.44	126.7	6.45	126.8
**C-18^{2}**	5.35 / 6.10	118.7	5.35 / 6.10	119.0
**C-19**		174.3		174.6
**C-20**	9.29	177.1	9.32	177.5
**C-1**		128.7		**129.2**
**C-2**		135.2		135.5
**C-2^{1}**	2.22	8.6	2.27	8.8
**C-3**		120.3		**121.3**
**C-3^{1}**	2.60-2.68	24.8	2.70	**25.4**
**C-3^{2}**	3.33-3.44 / 3.76	70.1	**3.45-3.53 / 3.63-3.69**	**71.6**
**C-4**		139.0		**138.3**
**C-5**	3.95 / 4.04	23.6	3.91 / 3.97	23.6
**C-6**		133.9		134.2
**C-7**		112.2		112.5
**C-7^{1}**	2.12	9.2	2.13	9.0
**C-8**		125.7		125.8
**C-8^{1}**				
**C-8^{2}**		67.7	3.78	68.1
**C-8^{3}**		171.2		171.6
**C-8^{5}**	3.76	52.5	3.76	52.7
**C-9**		160.9		160.9
**C-10**	4.88	36.9	4.88	**38.1**
**C-11**		124.2		124.5
**C-12**		120.1		119.8
**C-12^{1}**	2.60-2.68 / 2.68-2.79	21.7	**2.40-2.47 / 2.60-2.68**	**19.8**
**C-12^{2}**	2.32	38.7	2.31-2.39	**36.1**
**C-12^{3}**		180.0		**174.4**
**C-13**		115.2		115.5
**C-13^{1}**	1.92	9.2	1.89	9.0
**C-14**		124.4		**123.9**
**C-15**	2.42 / 2.88	30.1	2.50 / 2.89	**30.1**
**C-1´**	4.19	103.7	4.23	**104.6**
**C-2´**	3.17	74.8	3.13	74.8
**C-3´**	3.33-3.44	77.7	3.35	77.8
**C-4´**	3.24-3.27	71.1	3.25	**71.6**
**C-5´**	3.24-3.27	77.5	**3.45-3.53**	**75.2**
**C-6´**	3.64 / 3.78-3.90	62.3	**3.97 / 4.56**	**64.7**

Table S2. ¹H,¹H-coupling constants of **4** (from 2-dimensional ¹H *J*-resolved NMR-spectra, CD_3OD, 600 MHz).[8]

coupling	*J* / Hz	*J* / Hz a)
$J_{1,2}$	7.9	7.8
$J_{2,3}$	9.1	9.5
$J_{3,4}$	ca. 9.5	9.5
$J_{4,5}$	ca. 9.5	9.5
$J_{5, 6a}$	2.3	2.8
$J_{5,6b}$	6.7	5.7
$J_{6a, 6b}$	12.3	12.8

a) ¹H,¹H- coupling constants of β-D-Glucopyranose in D_2O [9]

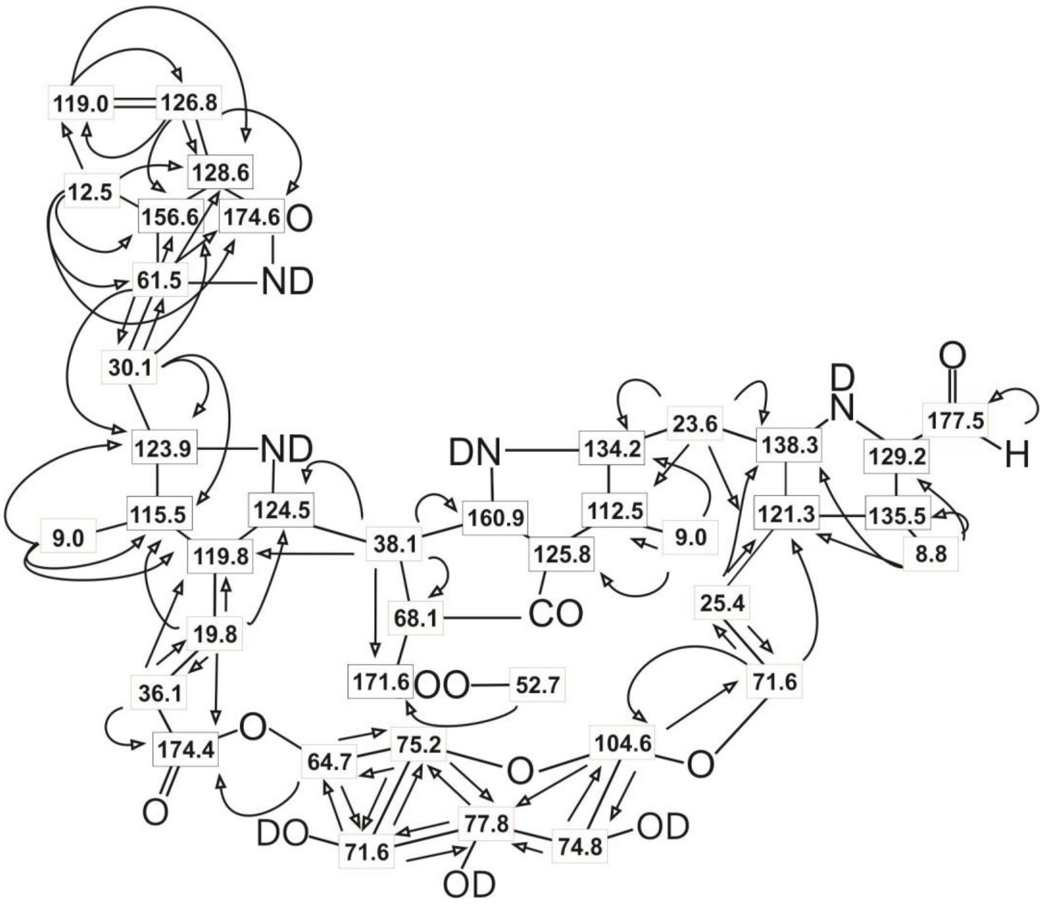

Figure S5. Heteronuclear single- and multiple-bond correlations (from ¹H,¹³C-HSQC and ¹H,¹³C-HMBC-spectra, respectively) and assignments of ¹³C-signals of *Ug*-NCC-53 (**4**).

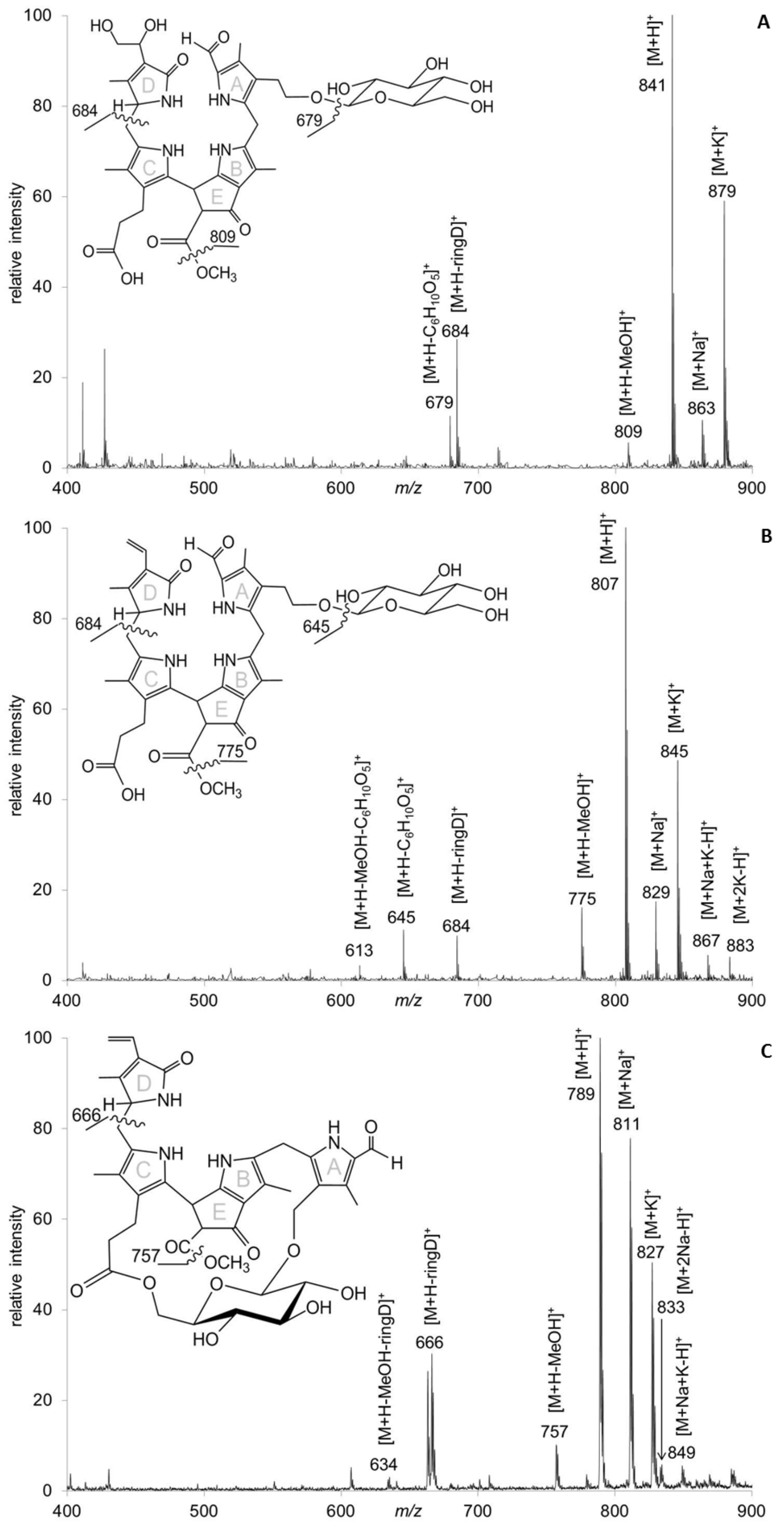

Figure S6. ESI mass spectra (on-line, positive-ion mode) of (A) *Ug*-NCC-27 (**2**), (B) *Ug*-NCC-43 (**3**) and (C) *Ug*-NCC-53 (**4**) (constitutional formulas are shown as inserts).

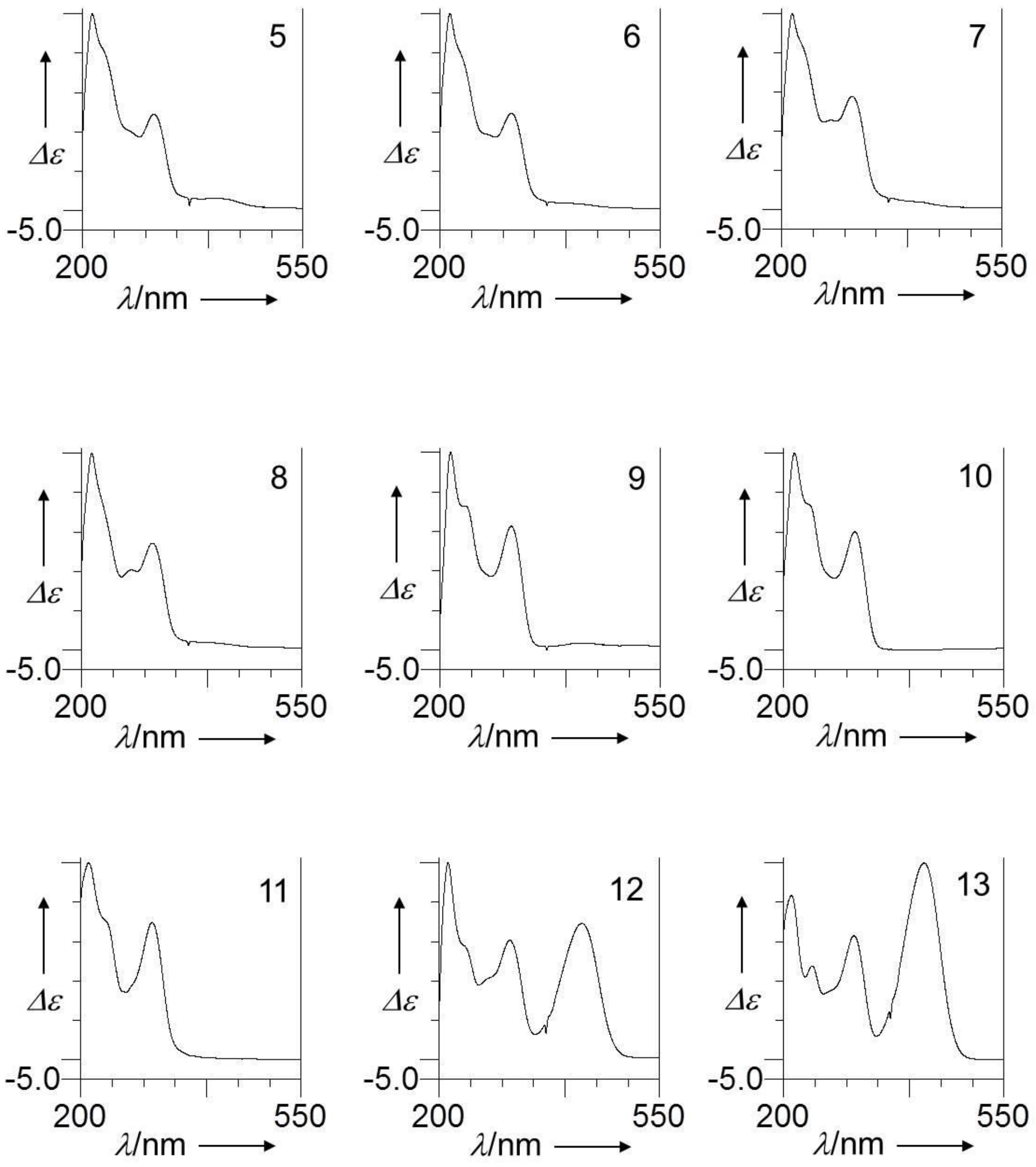

Figure S7. HPLC-analysis with on-line detection at 320 nm of an extract of senescent *Ulmus glabra* leaves (see Figure S2B): on-line UV/Vis-spectra of NCCs **5-11** and of the yellow chlorophyll catabolites (YCCs) **12** and **13**.

***Ug*-NCC-33 (5).** t_R=32.9 min. MS (ESI): *m/z* (%) = 883.27 (10, $[M + K + Na - H]^+$); 863.20 (23), 862.27 (52), 861.27 (100, $[M + K]^+$), 845.27 (80, $[M + Na]^+$); 823.07 (60, $[M + H]^+$; $C_{41}H_{51}N_4O_{14}^+$); 805.13 (65, $[M + H - H_2O]^+$); 700.00 (60, $[M + H - C_7H_9NO$ (ring D)$]^+$); 538.00 (45, $[M + H - C_6H_{10}O_5$ (sugar moiety) - C_7H_9NO (ring D)$]^+$; 487.20 (25, $[M + H – C_7H_9NO$ (ring D) – $C_9H_{11}NO_3$ (ring C) $-CH_3OH]^+$. MS/MS of the isolated ion at *m/z* 845.27 ($[M + Na]^+$): *m/z* (%) = 827.20 (12, $[M + Na - H_2O]^+$); 813.20 (100, $[M + Na - CH_3OH]^+$); 801.27 (8, $[M + Na - CO_2]^+$); 795.27 (2, $[M + Na – H_2O – CH_3OH]^+$); 722.27 (90, $[M + Na - C_7H_9NO$ (ring D)$]^+$); 690.20 (15, $[M + Na - C_7H_9NO$ (ring D)$-CH_3OH]^+$); 650.27 (8, $[M + Na - C_6H_{11}O_5 - CH_3OH]^+$.

***Ug*-NCC-37 (6).** t_R= 36.7 min. MS (ESI): *m/z* (%) = 877.27 (23), 876.27 (45), 875.20 (100, $[M + K]^+$), 859.27 (30, $[M + Na]^+$); 837.07 (25, $[M + H]^+$; $C_{42}H_{53}N_4O_{14}^+$); 805.20 (80, $[M + H - CH_3OH]^+$); 773.27 (3, $[M + H - CH_3OH - CH_3OH]^+$); 714.27 (52, $[M + H - C_7H_9NO$ (ring D)$]^+$); 682.27 (3, $[M + H - C_7H_9NO$ (ring D)$-CH_3OH]^+$); 643.20 (5, $[M + H-C_6H_{10}O_5$ (sugar moiety) - $CH_3OH]^+$); 611.27 (2, $[M+H-C_6H_{10}O_5$ (sugar moiety) - $CH_3OH - CH_3OH]^+$). MS/MS of the isolated ion at *m/z* 837.07: *m/z* (%) = 805.13 (100, $[M + H - CH_3OH]^+$); 714.27 (30, $[M + H - C_7H_9NO$ (ring D)$]^+$); 675.00 (2, $[M + H – C_6H_{10}O_5$ (sugar moiety)$]^+$.

***Ug*-NCC-38 (7).** t_R= 37.7 min. MS (ESI): *m/z* (%) = 845.27 (25, $[M + K]^+$); 829.33 (15, $[M + Na]^+$); 809.20 (13), 808.27 (80), 807.27 (100, $[M + H]^+$; $C_{41}H_{51}N_4O_{13}^+$); 775.27 (10, $[M + H - CH_3OH]^+$); 684.20 (5, $[M + H - C_7H_9NO$ (ring D)$]^+$); 645.20 (5, $[M + H - C_6H_{10}O_5$ (sugar moiety)$]^+$).

***Ug*-NCC-39 (8).** t_R= 39.2 min. MS (ESI): *m/z* (%) = 861.27 (11, $[M + K]^+$); 845.40 (18, $[M + Na]^+$); 825.13 (15), 824.22 (50), 823.20 (100, $[M + H]^+$; $C_{41}H_{51}N_4O_{14}^+$); 791.33 (5, $[M + H - CH_3OH]^+$).

***Ug*-NCC-48 (9).** t_R= 48.3 min. MS (ESI): *m/z* (%) = 1007.33 (55, $[M + K]^+$); 991.33 (28, $[M + Na]^+$); 970.13 (48), 969.20 (100, $[M + H]^+$, $C_{47}H_{61}N_4O_{18}^+$); 809.20 (13); 951.20 (4, $[M + H - H_2O]^+$); 807.27 (62, [M + H – $C_6H_{10}O_5$ (sugar moiety)]$^+$); 775.4 (5, [M + H - $C_6H_{10}O_5$ (sugar moiety) - CH_3OH]); 684.27 (2, [M + H - $C_6H_{10}O_5$ (sugar moiety) - C_7H_9NO (ring D)]$^+$); 652.33 (5, [M + H - $C_6H_{10}O_5$ (sugar moiety) - C_7H_9NO (ring D)-CH_3OH]$^+$); 645.20 (5, [M + H – $C_6H_{10}O_5$ – $C_6H_{10}O_5$ (sugar moiety)]$^+$); 608.13 (5, [M + H - $C_6H_{10}O_5$ (sugar moiety) - C_7H_9NO (ring D)-CH_3OH-CO_2]$^+$). MS/MS of the isolated ion at *m/z* 969.20: *m/z* (%) = 951.20 (11, $[M + H - H_2O]^+$); 937.20 (26, $[M + H - CH_3OH]^+$); 919.13 (2, $[M + H - CH_3OH - H_2O]^+$); 846.27 (1, [M + H - C_7H_9NO (ring D)]$^+$); 807.20 (62, [M + H – $C_6H_{10}O_5$ (sugar moiety)]$^+$); 789.20 (2, $[M + H – C_6H_{12}O_6]^+$); 775.13 (4, [M + H – $C_6H_{10}O_5$ (sugar moiety) - CH_3OH]$^+$); 645.07 (4, [M+H – $C_6H_{10}O_5$ – $C_6H_{10}O_5$ (sugar moiety)]$^+$). HR-MALDI-TOF-MS: m/z_{found} = 969.3945 ($[M + H]^+$), m/z_{calc} ($C_{47}H_{61}N_4O_{18}^+$) = 969.3975.

***Ug*-NCC-50 (10).** t_R= 49.6 min. MS (ESI): *m/z* (%) = 1099.40 (30, $[M+K]^+$); 1083.40 (25, $[M+Na]^+$); 1063.33 (24), 1062.27 (60), 1061.27 (100, $[M+H]^+$, $C_{53}H_{65}N_4O_{19}^+$), 1029.33 (4, $[M + H – CH_3OH]^+$); 951.20 (8, $[M + H – C_6H_6O_2]^+$; 899.27 (3, [M + H – $C_6H_{10}O_5$ (sugar moiety)]$^+$); MS/MS of the isolated ion at *m/z* 1061.27: *m/z* (%) = 1043.33 (1, $[M + H – H_2O]^+$; 1029.27 (100, $[M + H – CH_3OH]^+$); 951.27 (30, $[M + H – C_6H_6O_2]^+$; 938.13 (5, [M + H - C_7H_9NO (ring D)]$^+$); 933.33 (0.7, $[M + H – H_2O - C_6H_6O_2]^+$; 919.27 (6, $[M + H – CH_3OH - C_6H_6O_2]^+$; 915.27 (4, $[M + H – H_2O – H_2O - C_6H_6O_2]^+$; 906.27 (1, [M + H – CH_3OH - C_7H_9NO (ring D)]$^+$); 899.20 (35, [M

+ H – $C_6H_{10}O_5$ (sugar moiety)]$^+$); 883.20 (1, [M + H – H_2O –H_2O – CH_3OH - $C_6H_6O_2$]$^+$; 867.13 (5, [M+H – $C_6H_{10}O_5$ (sugar moiety) – CH_3OH]$^+$); 789.07 (5, [M + H - $C_6H_{10}O_5$ (sugar moiety) - $C_6H_7O_2$]$^+$; HR-MALDI-TOF-MS: m/z_{found} = 1061.4247 ([M + H]$^+$), m/z_{calc} ($C_{53}H_{65}N_4O_{19}^+$) = 1061.4238.

***Ug*-NCC-57 (11).** t_R= 56.8 min. MS (ESI): *m/z* (%) = 859.33 (75, [M + K]$^+$); 843.33 (35, [M + Na]$^+$); 823.27 (23), 822.27 (48), 821.20 (100, [M + H]$^+$; $C_{42}H_{53}N_4O_{13}^+$); 789.27 (65, [M + H - CH_3OH]$^+$); 757.33 (10, [M + H - CH_3OH - CH_3OH]$^+$); 698.20 (15, [M+H - C_7H_9NO (ring D)]$^+$); 666.27 (8, [M+H – CH_3OH - C_7H_9NO (ring D)]$^+$; 659.27 (15, [M + H - $C_6H_{10}O_5$ (sugar moiety)]$^+$); 627.27 (8, [M + H – CH_3OH - $C_6H_{10}O_5$ (sugar moiety)]$^+$); MS/MS of the isolated ion at *m/z* 821.20: *m/z* (%) = 789.20 (100, [M + H - CH_3OH]$^+$); 698.20 (5, [M + H - C_7H_9NO (ring D)]$^+$); 659.27 (30, [M + H - $C_6H_{10}O_5$ (sugar moiety)]$^+$); 627.20 (5, [M+H-$C_6H_{10}O_5$ (sugar moiety) - CH_3OH]$^+$).

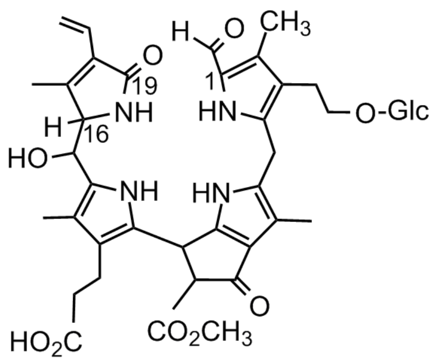

Ug-NCC-33 (**5**) & *Ug*-NCC-39 (**8**)

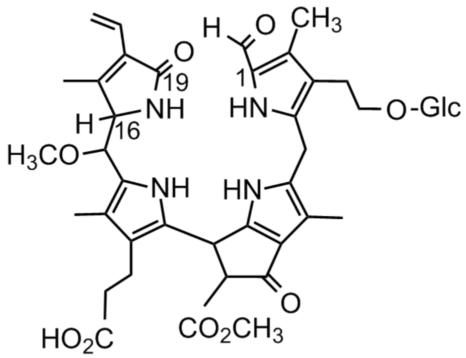

Ug-NCC-37 (**6**)

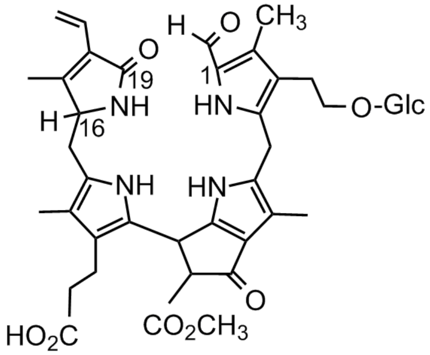

Ug-NCC-38 (**7**), isomer of **3**

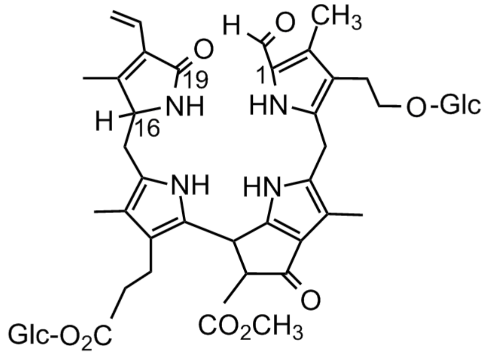

Ug-NCC-48 (**9**)

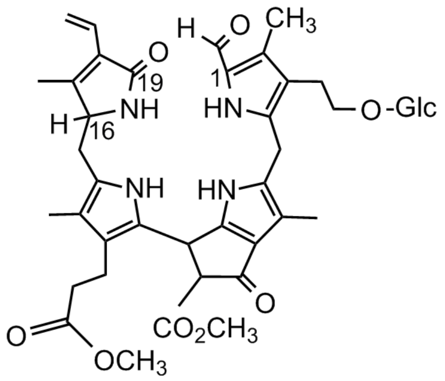

Ug-NCC-57 (**11**)

Figure S8. Tentative constitutional formulas of NCCs **5** – **9** and **11**, derived from UV/Vis- and mass spectral data.

Identification of Ug-NCC-43 (3) and Tc-NCC-2 (from Lime tree) using HPLC. A yellow Wych Elm tree leaf and (in a second experiment) a yellow Lime tree leaf were ground in a mortar with 0.2 g of sea sand, frozen with liquid N_2 and extracted with about 2 ml of MeOH. The suspension was each centrifuged at 13,000 rpm for 5 min. The clear supernatant (200 µl) was diluted with 200 µl aq. potassium phosphate buffer solution ($pH = 7$) and again centrifuged at 13,000 rpm for 5 min. Separated samples of *Ug*-NCC-43 (**3**) and of *Tc*-NCC-2 [10], as well as a 1:1 mixture of **3** and *Tc*-NCC-2 were analyzed by HPLC. *Ug*-NCC-43 (**3**) and *Tc*-NCC-2 eluted both at a t_R of about 31 min.

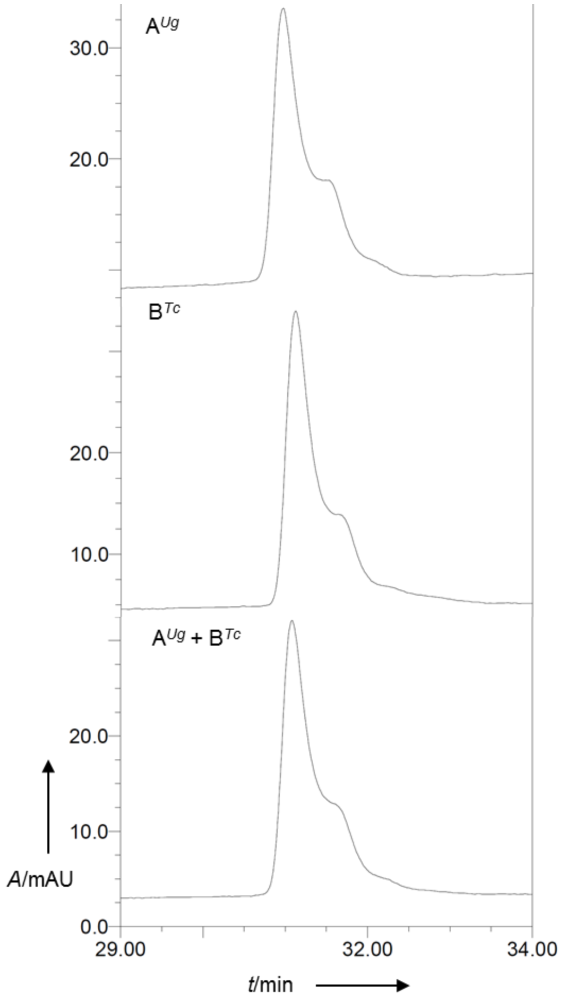

Figure S9. Identification of *Ug*-NCC-43 (A^{Ug}) and of *Tc*-NCC-2 (B^{Tc}) by analytical HPLC, including a co-injection experiment.

Epimerization of *Ug*-NCC-53 (4).

A purified sample of *Ug*-NCC-53 (**4**), in addition to the main fraction, contained a more polar fraction (*ca*. 7 %). This minor fraction, named *Ug*-NCC-51, was presumed to be an epimer of **4**, due to epimerization at C(8^2) of the β-keto ester functionality. Both fractions were separated by HPLC (injection volume: 0.1 ml, flow rate: 0.5 ml min^{-1}; solvent composition see section final purification of **4**) and collected separately. The minor fraction was stored for an hour at room temperature dissolved in a 2:1 mixture of 10 mM ammonium acetate (*pH* 7.0) and acetonitrile. From reanalysis of a sample of this fraction by HPLC two NCC-fractions *Ug*-NCC-51 and *Ug*-NCC-53 were again observed in a roughly 1:1 ratio, as shown below.

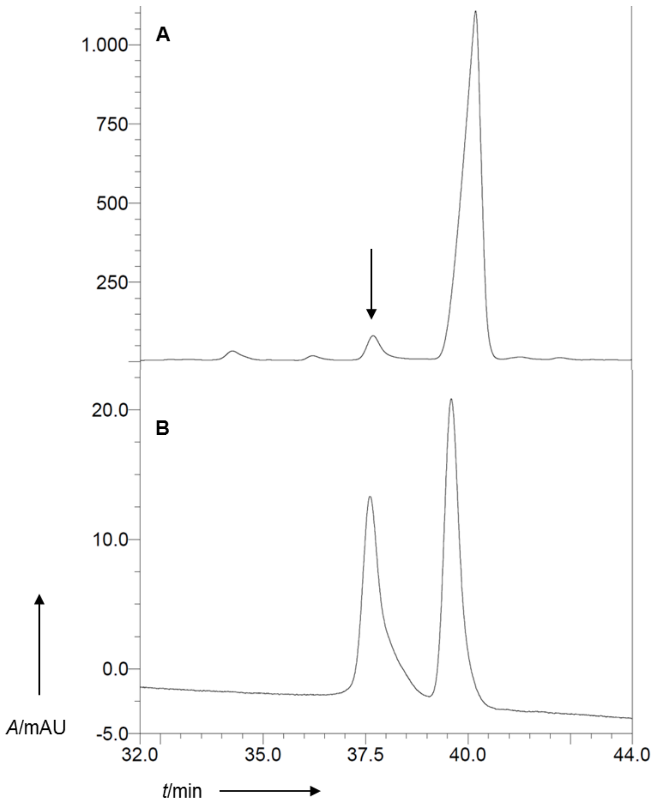

Figure S10. HPLC-analysis of the epimerization of *Ug*-NCC-53 (**4**). A: Reanalysis of an isolated fraction of **4**, B: Reanalysis of the separated fraction of the more polar, minor isomer (highlighted with an arrow in A).

Molecular Dynamics Simulations (see main text for details)

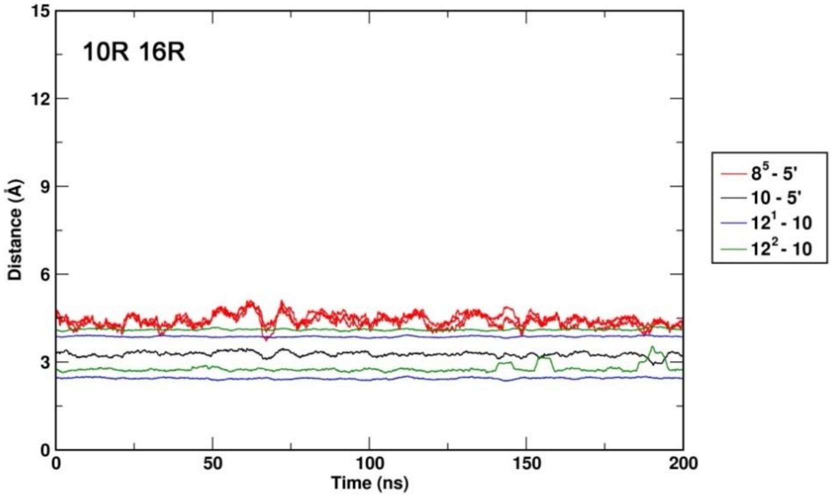

Figure S11. Molecular dynamics simulation (200 nanoseconds of sampling) using explicit solvent molecular dynamics of the modelled (10R,16R)-stereoisomer of the NCC **4**. Distances of atom-pairs H_3C8^5-HC5´, HC10-H5', HC10-$H_{A/B}C12^1$, HC10-$H_{A/B}C12^2$ were computed.

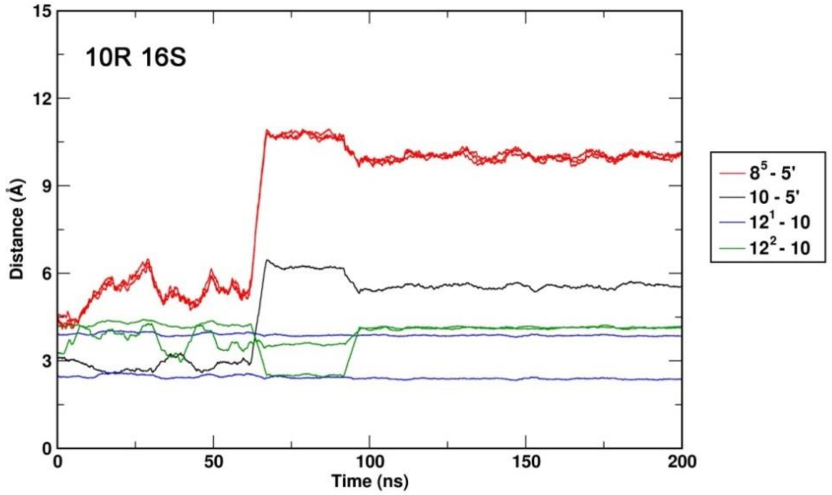

Figure S12. Molecular dynamics simulation (200 nanoseconds of sampling) using explicit solvent molecular dynamics of the modelled (10R,16S)-stereoisomer of the NCC **4**. Distances of atom-pairs H_3C8^5-HC5´, HC10-H5', HC10-$H_{A/B}C12^1$, HC10-$H_{A/B}C12^2$ were computed.

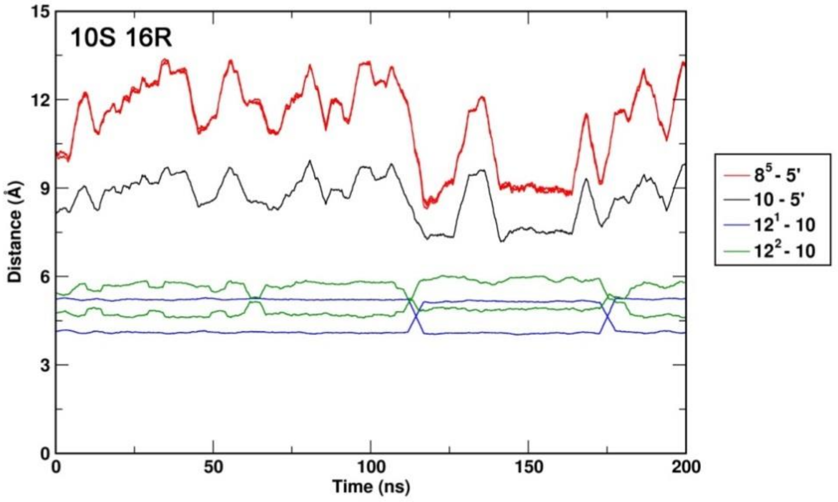

Figure S13. Molecular dynamics simulation (200 nanoseconds of sampling) using explicit solvent molecular dynamics of the modelled (10S,16R)-stereoisomer of the NCC **4**. Distances of atom-pairs H_3C8^5-HC5´, HC10-H5’, HC10-$H_{A/B}C12^1$, HC10-$H_{A/B}C12^2$ were computed.

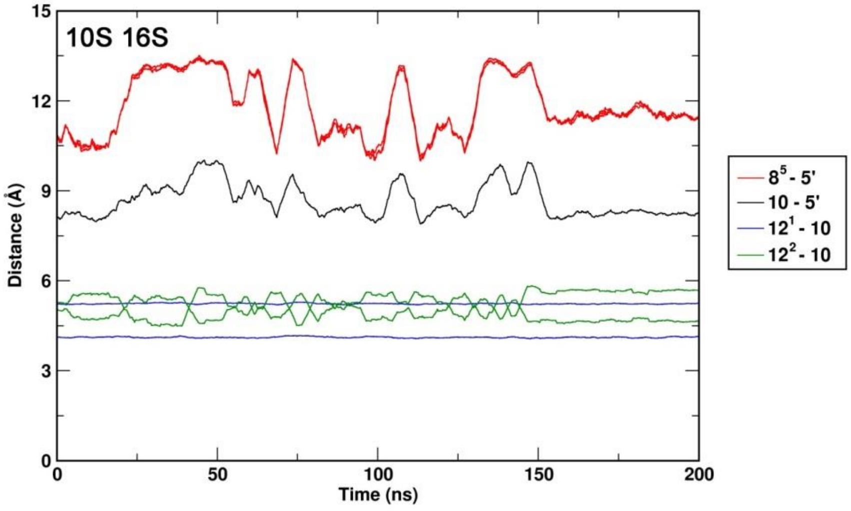

Figure S14. Molecular dynamics simulation (200 nanoseconds of sampling) using explicit solvent molecular dynamics of the modelled (10S,16S)-stereoisomer of the NCC **4**. Distances of atom-pairs H_3C8^5-HC5´, HC10-H5’, HC10-$H_{A/B}C12^1$, HC10-$H_{A/B}C12^2$ were computed.

Substructure Searches

The basic structure queries used in *SciFinder* (https://scifinder.cas.org) [11] and *Reaxys* (https://www.reaxys.com) [12] are shown below (Figure **S15**). In order to include macrocyclic rings of the desired size in the queries, the *repeating group/atom* feature was used in both *SciFinder* and *Reaxys*, with **A** = any atom (except for H-atoms) and any bond type (dotted lines). Any substitution as well as annulation of further rings were permitted anywhere in the query structure.

1,2 bridged

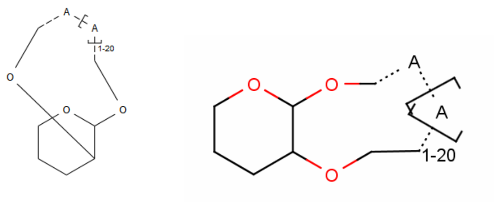

1,3 bridged

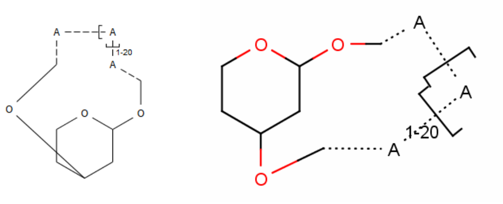

1,4 bridged

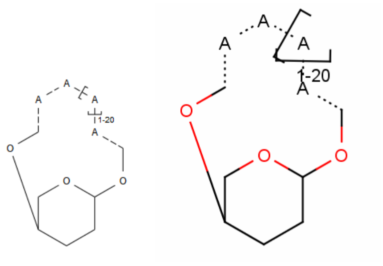

1,5 bridged

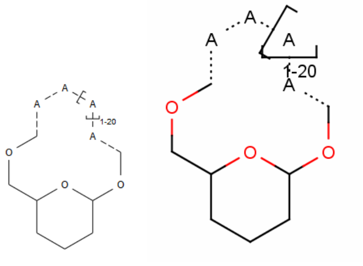

2,3 bridged ≅ 3,4 bridged

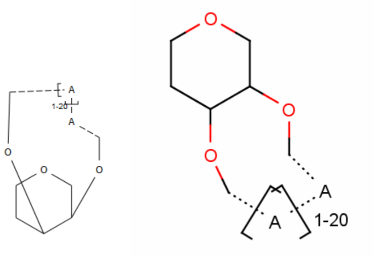

2,4 bridged

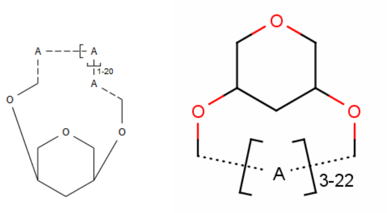

2,5 bridged

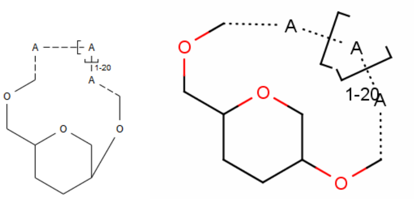

3,5 bridged

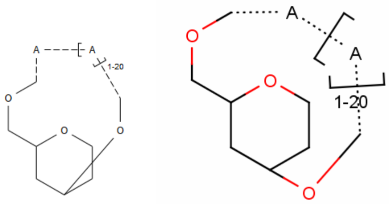

4,5 bridged

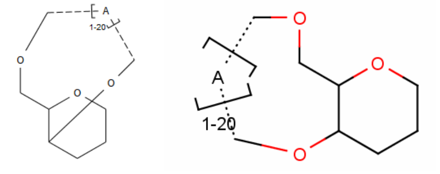

1,6-Hexopyranoses

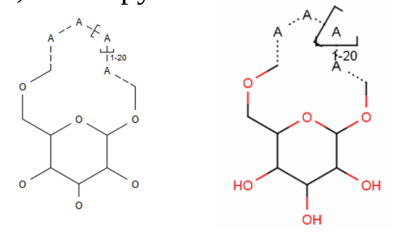

1,6-Glucopyranose-lacton

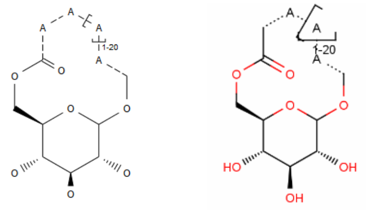

Figure S15. Substructure queries (screen dumps) used for the different macrocycle-bridged pyranoses in CAS *SciFinder* (left) and Elsevier *Reaxys* (right). As a first restriction to these macrocyclic lactones, a carbonyl group was added at the higher-numbered attachment point of the pyranose ring. Further restrictions to compounds possessing at least one N atom were achieved by the "Analyze by: Elements" feature in *SciFinder*, and with "Filter by Substructure" in *Reayxs* using a single N-atom as query. The final structural restriction was done by using a ring with 4 C and 1 N-atom and bonds of any type in both database systems. These searches gave the results shown in Table **S3**.

Table S3. Macrocycle-bridged pyranoses in CAS *SciFinder* and Elsevier *Reaxys*: number of compound records retrieved (numbers in parentheses: compounds classified as *natural products*, see text); refs = number of references retrieved

**Bridge**	**Database**	**Compounds**	**+ Lactone**	**+ N-containing.**	**+ C5N ring**
1,2	SciFinder	3‘611	412 (130)	34 (0)	10 (0) [3 refs]
	Reayxs	2‘256 (597)	479 (204)	33 (0)	10 (0) [3 refs]
1,3	SciFinder	871	372 (96)	18 (0)	0 (0)
	Reayxs	675 (201)	470 (160)	17 (0)	0 (0)
1,4	SciFinder	9‘299	123 (2)	3 (0) [2 refs]	0 (0)
	Reayxs	518 (32)	41 (1)	1 (0)	0 (0)
1,5	SciFinder	1‘135	474 (61)	107	14 (0) [8 refs]
	Reayxs	927 (104)	482 (74)	86 (0)	14 (0) [8 refs]
2,3 =3,4	SciFinder	2‘320	613 (41)	125 (0)	7 (0) [6 refs]
	Reayxs	2‘185 (279)	730 (224)	55 (0)	9 (0) [6 refs]
2,4	SciFinder	779	307 (45)	66 (0)	4 (0) [1 ref]
	Reayxs	553 (148)	500 (145)	71 (1)	2 (0) [1 ref]
2,5	SciFinder	375	29 (1)	1 (0) [1 ref]	0 (0)
	Reayxs	526 (58)	69 (24)	8 (0)	2 (0) [1 ref]
3,5	SciFinder	473	359 (41)	94 (0)	3 (0) [1 ref]
	Reayxs	686 (162)	596 (162)	104 (1)	3 (0) [2 refs]
4,5	SciFinder	2‘002	704 (119)	86 (0)	2 (0) [2 refs]
	Reayxs	2‘637 (503)	1‘276 (463)	65 (0)	0 (0)
**TOTAL**	**SciFinder**	**17‘692 (630)**	**2‘748 (458)**	**413 (0)**	**31 (0) [12 refs]**
	**Reayxs**	**7‘849 (1‘463)**	**3‘586 (1‘104)**	**321 (1)**	**32 (0) [12 refs]**

Of the bridged macrocycles containing a five-membered ring with an N-atom and thus most closely related structurally to the catabolite **4** - *SciFinder*: 31, *Reayxs*: 32 (cf. column "+C4N" in Table **S3**) - 26 were found in both database systems. One of the five compounds retrieved exclusively in *SciFinder* was a false hit, a two-component system with the pyrrole ring in one, and the bridged pyranose in the other component (this compound was consequently not retrieved in *Reaxys*, and could not have been retrieved anyway as the corresponding publication is not present there). The other four compounds exclusively in *SciFinder* were missed by *Reayxs* because they had not been indexed from the two respective publications present also in *Reaxys*. Regarding the six compounds retrieved in *Reaxys* but not in *SciFinder*, two had not been indexed by CAS (*Chemical Abstracts Service*) for a publication otherwise covered; the other four compounds did only appear as products of reactions which had been indexed for the two respective publications in *Reaxys*, but not by CAS. - A total of 14 references were retrieved for these compounds (8 for the 1,5-bridged macrocycles), 10 common to both sources, and two each exclusively in *SciFinder* and *Reayxs*, respectively. Of the two references retrieved exclusively in *SciFinder* but missed in *Reaxys*, one was missing in the latter database, for the other reference present, the relevant compound had not been indexed in *Reaxys*. The two references retrieved exclusively in *Reaxys* were both present in *SciFinder*, but had the respective relevant compounds not indexed, and were thus not retrieved. These differences in our search results, as many other similar examples, exemplify the necessity to search BOTH large compound databases.

The only "natural product" retrieved (cf. Table **S3**) among macrocyclic lactones containing at least one nitrogen was found to be actually only a synthetic derivative of the nitrogen-free natural product Aleurinin [13]. Such a restriction to *natural products* had to be done quite differently in the two database systems involved: in *Reayxs*, this information is placed in a specific data field "Isolation from Natural Product", and "Filter by: Natural Product" allows a simple and precise restriction to such compounds. In the literature database CAPLUS, natural products are in principle indexed with the substance role [14] "natural product occurrence"; this precise role, however, is not directly searchable in *SciFinder* (in contrast to another user interface to the same CAS databases, STN *Messenger* [15], not available to us). One can search only for the more general substance role "Occurrence" to determine the numbers given in Table S3; as this broader role is also used for occurrence in the environment (e.g., pollutants), or in analytical samples, such results may give too high a number.

Unfortunately, this substance indexing, or the related indexing for the entire publication record with CA *General Subject Heading* like "Molecular structure, natural product", "New Natural products", "Nomenclature, new natural products", and "Pharmaceutical natural products", does not nearly cover the entire time range of the CAPLUS database (in our cases, only back to about 1967). As a consequence, for precise results, ALL publication records for compounds in question must be printed out (or exported) and inspected manually to reliably identify natural products by their indexing with telltale modifying phrases like "from genus species". This tedious and very time consuming procedure in the example of hexofuranoses bridged in positions 1,6 with a macrocylic lactone (cf. Table **S4**) confirmed all 60 compounds retrieved with substance role "occurrence" as natural products (with 63 relevant references going back only to 1990), but we found no less than 71 additional natural products with 40 additional references (13 before 1990) among the remaining 408 macrocycles not assigned the role "occurrence". For the 532 1,6-bridged hexoses **not** containing a macrocyclic lactone (difference between columns “Compounds” and “+ Lactone” in Table **S4**), only 14 of the 19 compounds with “Occurrence” were considered as natural products, while an additional seven natural products were identified lacking this role.

Table S4. Bicyclo[n.3.1]glycoside and –glucoside compound records retrieved in *CAS SciFinder* (numbers in parentheses: compounds with substance role “occurrence”)

**Substructure**	**Compounds**	**+ Lactone**	**+ N-containing**	**+ C5N ring**
**Hexo**pyranoses	980 (79)	468 (60)	107 (0)	14 (0) [8 refs]
**D-Gluco**pyranoses	693 (69)	323 (52)	67 (0)	6 (0) [2 refs]
**Non-D-gluco**	148 (3)	67 (1)	15 (0)	2 (0) [4 refs]
**No stereo…**	139 (7)	78 (7)	25 (0)	6 (0) [2 refs]

Another database difference had to be taken into account in the searches for bridged *D-gluco*pyranose derivatives involving chiral query structures (cf. Figure **S11**): in *Reayxs*, the desired configuration (without specifying the anomeric center, i.e. retrieving both α- and β-anomers) was specifically searched for; the category "Other Hexopyranoses" in Table **S4** was determined as the resulting hitset from "Hexopyranoses NOT Glucopyranoses". In *SciFinder*, from the stereo analysis automatically invoked with all chiral query structures, the hit categories "Absolute stereo match", "Stereo that doesn't match query" (which contains all diastereomers of D-glucose: "Non-D-gluco" in Table **S4**), and "No stereo in answer structure" were examined.

This latter category contains predominantly compounds registered at a time when earlier versions of the *CAS Registry System* [16] could not yet handle the configuration of compounds in the structure, but only via stereodescriptors in the compound name, i.e. "gluco", "glucopyranose" [17]. The systematic names of all compounds thus retrieved had then to be checked for the stereodescriptor "gluco".

Table S5. Bicyclo[n.3.1]glycoside and –glucoside structures retrieved in *Elsevier Reaxys* (numbers in parentheses: compounds with "Isolation from Natural Product")

**Substructure**	**Compounds**	**+ Lactone**	**+ N-containing**	**+ C5N ring**
**Hexo**pyranoses	841 (99)	479 (73)	86 (0)	14 (0) [8 refs]
**D-Gluco**pyranoses	654 (83)	342 (62)	68 (0)	10 (0) [4 refs]
**Other Hexopyranoses**	187 (16)	137 (11)	18 (0)	4 (0) [4 refs]

Under the category "Other Hexopyranoses" in Table **S5** also bridged **L**-glucoses were retrieved: a total of 48 such compounds, 32 with a lactone bridge (13 were listed as natural products), but none containing N. In *SciFinder* (see Table **S4**), these compounds show up in "Non-D-gluco" (four, all lactones, none with N, no natural products) and "No stereo given" (none).

References

[1] B. Kräutler, *Angew. Chem. Int. Ed.* **2016**, *DOI:10.1002/anie.201508928R1*.
[2] H. E. Gottlieb, V. Kotlyar, A. Nudelman, *J. Org. Chem.* **1997**, *62*, 7512-7515;
[3] L. Ernst, *J. Chem. Soc. - Perkin Trans. 1* **1984**, 2267-2270.
[4] H. Kessler, M. Gehrke, C. Griesinger, *Angew Chem Int Ed* **1988**, *27*, 490-536.
[5] R. J. Porra, W. A. Thompson, P. E. Kriedemann, *Biochim. Biophys. Acta* **1989**, *975*, 384-394.
[6] C. Curty, N. Engel, *Phytochem.* **1996**, *42*, 1531-1536.
[7] B. Kräutler, *Chem. Soc. Rev.* **2014**, *43*, 6227-6238.
[8] G. Wider, K. Wüthrich, *J Magn Reson Ser B* **1993**, *102*, 239-241.
[9] E. Pretsch, P. Bühlmann, C. Affolter, *Structure Determination of Organic Compounds*, Springer Verlag, Berlin, **2000**.
[10] M. Scherl, T. Müller, B. Kräutler, *Chem. Biodiv.* **2012**, *9*, 2605 - 2617.
[11] https://scifinder.cas.org; http://www.cas.org/products/scifinder (last accessed Apr. 7th, 2016).
[12] https://www.reaxys.com; https://www.reaxys.com/documentation/about_query.shtml; https://www.elsevier.com/solutions/reaxys (last accessed Apr. 7th, 2016).
[13] G. Nonaka, M. Hayashi, T. Tanaka, R. Saijo, and I. Nishioka, *Chem. Pharm. Bull.* **1990**, *38*, 861-865.
[14] https://www.cas.org/File%20Library/Training/STN/User%20Docs/casroles.pdf (last accessed March 22nd, 2016).
[15] https://www.cas.org/products/stn/user-resources (last accessed March 22nd, 2016).

[16] D. W. Weisgerber, *J. Am. Soc. Inf. Sci.* **1997**, *48*, 349-360.
[17] J. E. Blackwood, P. E. Blower jr., S. W. Layten, D. H. Lillie, A.H. Lipkus, J. P. Peer, C. Qian, L. M. Staggenborg and C. E. Watson, *J. Chem. Inf. Comput. Sci.* **1991**, *31*, 204-212.
